# Fast4DReg – fast registration of 4D microscopy datasets

**DOI:** 10.1242/jcs.260728

**Published:** 2023-02-27

**Authors:** Joanna W. Pylvänäinen, Romain F. Laine, Bruno M. S. Saraiva, Sujan Ghimire, Gautier Follain, Ricardo Henriques, Guillaume Jacquemet

**Affiliations:** ^1^ Åbo Akademi University, Faculty of Science and Engineering, Biosciences, Turku 20520, Finland; ^2^ Turku Bioimaging, University of Turku and Åbo Akademi University, Turku 20520, Finland; ^3^ Turku Bioscience Centre, University of Turku and Åbo Akademi University, Turku 20520, Finland; ^4^ MRC Laboratory for Molecular Cell Biology, University College London, London WC1E 6BT, UK; ^5^ The Francis Crick Institute, London NW1 1AT, UK; ^6^ Instituto Gulbenkian de Ciência, Oeiras 2780-156, Portugal; ^7^ InFLAMES Research Flagship Center, Åbo Akademi University, Turku 20520, Finland

**Keywords:** 3D drift correction, Live imaging, Image analysis, ImageJ, Fiji

## Abstract

Unwanted sample drift is a common issue that plagues microscopy experiments, preventing accurate temporal visualization and quantification of biological processes. Although multiple methods and tools exist to correct images post acquisition, performing drift correction of three-dimensional (3D) videos using open-source solutions remains challenging and time consuming. Here, we present a new tool developed for ImageJ or Fiji called Fast4DReg that can quickly correct axial and lateral drift in 3D video-microscopy datasets. Fast4DReg works by creating intensity projections along multiple axes and estimating the drift between frames using two-dimensional cross-correlations. Using synthetic and acquired datasets, we demonstrate that Fast4DReg can perform better than other state-of-the-art open-source drift-correction tools and significantly outperforms them in speed. We also demonstrate that Fast4DReg can be used to register misaligned channels in 3D using either calibration slides or misaligned images directly. Altogether, Fast4DReg provides a quick and easy-to-use method to correct 3D imaging data before further visualization and analysis.

## Teaser

**Summary:** Fast4DReg is an open-source Fiji plugin that can quickly correct axial and lateral drift in multidimensional microscopy datasets.

## Introduction

Live imaging is essential in biomedical research, enabling scientists to follow biological processes over time. Despite being heavily used, performing live-imaging experiments using fluorescence microscopy remains technically challenging. The user must carefully balance illumination power and acquisition speed while maintaining specimen health. In addition, imaging is often prone to drift. Drift can be caused, for example, by temperature changes leading to thermal expansion of the microscope mechanical components or by the movement of the sample itself.

Multiple software and hardware solutions have been developed to minimize drifting during acquisition. For instance, axial drifting can be limited using an infrared light that is reflected on the glass-sample interface and captured by a detector (e.g. Leica's Adaptive Focus Control or Nikon's Perfect Focus System). Lateral drift due to sample movement can also be compensated by tracking algorithms that follow the sample over time and move the microscope stage accordingly (Fox et al., 2022; von Wangenheim et al., 2017). Yet, drifting is rarely entirely eliminated at the acquisition stage, especially when acquiring multiple positions for an extended period. Therefore, it is often necessary to perform drift correction (via image registration) as a post-processing step before image visualization and quantification. Beyond live imaging, drift correction and/or channel registration is a crucial processing step for multiple image-analysis pipelines, including colocalization analysis (using calibration slides) or the reconstruction of super-resolution microscopy images.

Most drift-correction/registration algorithms work sequentially by comparing a reference image to a moving image and estimating the movement between these two images to correct the drift. Multiple open-source tools capable of correcting four-dimensional (4D) datasets already exist. Popular tools include, for instance, Insight ToolKit (McCormick et al., 2014), elastix (Klein et al., 2010), Multiview Reconstruction (Preibisch et al., 2010; 2014), Fijiyama (Fernandez and Moisy, 2021) or Correct 3D drift (Correct3DD) (Parslow et al., 2014). However, except for Multiview Reconstruction and Correct3DD, these tools are geared toward correcting medical imaging datasets and can be impractical to use for the correction of long three-dimensional (3D) videos. Multiview Reconstruction, which was designed to register large light-sheet fluorescence microscopy datasets, uses interest points (e.g. fluorescent beads, nuclei or membrane markers) in the imaging volume to perform the 3D registration, which are not always available (Preibisch et al., 2010; 2014). Although we routinely use Correct3DD, we felt limited by its speed and available features.

Here, prompted by a need to correct our 3D videos more easily and more efficiently, we developed Fast4DReg, a fast two-dimensional (2D) and 3D video drift-correction tool. Using multiple datasets, we show that Fast4DReg can outperform two state-of-the-art 3D video drift-correction tools available in Fiji, namely, Correct3DD (Parslow et al., 2014) and Fijiyama (Fernandez and Moisy, 2021). In addition, we show that Fast4DReg can register misaligned channels in 3D using either calibration slides or misaligned images directly. Fast4DReg is fast and has an easy-to-use graphical interface. These features make Fast4DReg a versatile and easy-to-use open-source 2D/3D drift-correction tool.

## RESULTS

### The Fast4DReg pipeline

**Fig. 1.**
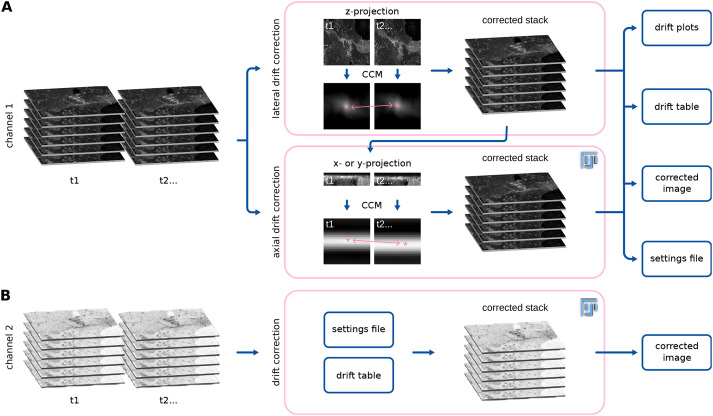
**Drift correction of 3D videos using Fast4DReg.** Scheme highlighting the Fast4DReg pipeline. (A) Fast4DReg sequentially estimates the lateral drift, corrects the lateral drift, and then estimates and corrects the axial drift. Fast4DReg creates intensity projections along multiple axes and estimates the drift between the reference and moving frames by calculating their cross-correlation matrix (CCM). The location of the peak intensity in the CCM (pink asterisk) defines the linear shift between the two images (as highlighted by the pink double-headed arrow). Fast4DReg outputs the corrected images, the drift plots, a drift table, and a settings file containing all selected parameters and paths to the drift table. (B) The settings file inducing the used parameters and path to the drift table can then be applied to correct other datasets (i.e. another channel) directly.

Fast4DReg breaks the drift-correction task into two steps: image registration (estimation of a transformation that corrects the drift optimally) followed by image transformation (applying the determined parameters to produce a corrected image). To estimate the drift of a 3D video in the *x-*, *y-* and *z-*coordinates, Fast4DReg sequentially estimates the lateral drift, corrects the lateral drift, then estimates and corrects the axial drift (Fig. 1). Lateral and axial drift corrections can also be performed independently, which can be particularly useful when only the axial drift needs to be corrected. As an output of the drift estimation step, Fast4DReg creates a new folder containing the corrected images, drift plots (graphs indicating the amount of drift detected), a drift table (drift detected in numerical values) and a settings file containing the selected parameters. Notably, the drift table can then be applied to correct other images using the same parameters (i.e. to correct another channel). Indeed, when correcting multichannel 3D videos, the user needs to choose one channel to use to estimate the drift. The other channel(s) can then be corrected using the same drift table (Fig. 1). To estimate the lateral or axial drift of a 3D video, Fast4DReg creates *z-* or *y*-intensity projections for each time point to create a 2D video. Fast4DReg then estimates the linear drift between the reference and moving frames by calculating their cross-correlation matrix (CCM) (see Materials and Methods for more details). The location of the peak intensity in the CCM defines the linear shift between the two images. Sub-pixel accuracy is accomplished by upscaling the CCM via bicubic spline interpolation (as demonstrated by Laine et al., 2019). Depending on their data, users can choose the first frame (best for fixed data) or consecutive frames from the movie (best for live-imaging data) as the reference frame.

### Fast4DReg outperforms Correct3DD or Fijiyama on our synthetic dataset

**Fig. 2.**
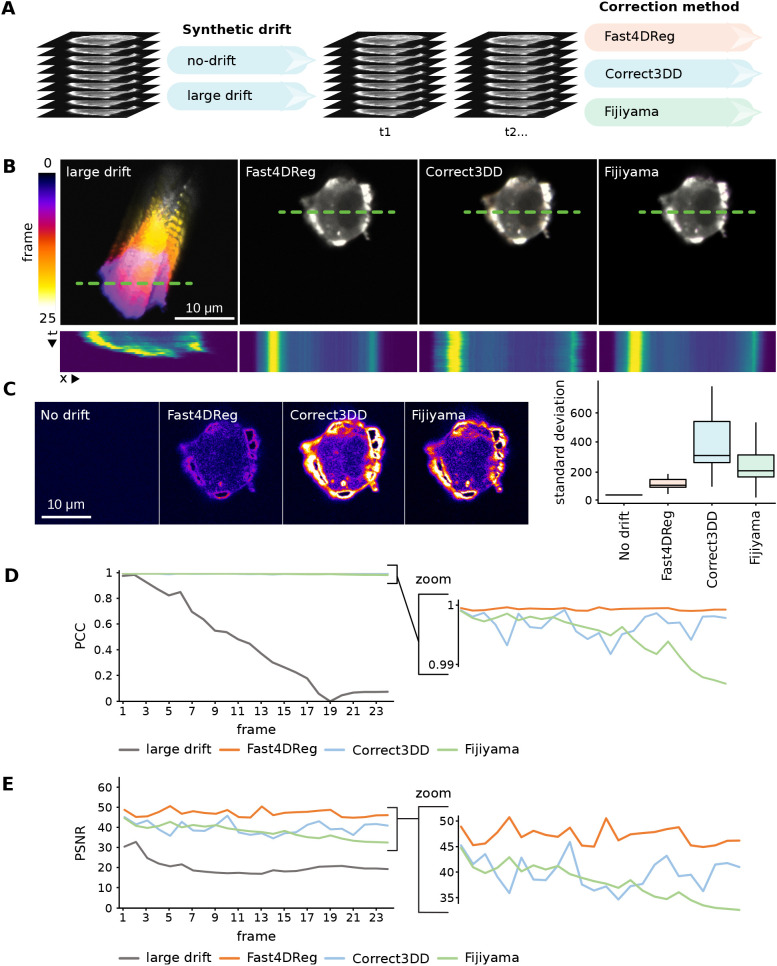
**Fast4DReg outperforms Correct3DD and Fijiyama on a synthetic dataset.** (A) Two synthetic 3D video datasets were created, one with no drift and another with a large amount of drift (see also Fig. S1). The large drift dataset was then corrected using Fast4DReg, Correct3DD and Fijiyama. (B) The drift-correction performance of the three algorithms was assessed using temporal color projections of a selected *z*-slice (middle of the cell) and kymographs (along the green dashed lines; dimensions, 25 μm × 25 frames). (C) Standard deviation time projection of the middle slice of the cell. This projection takes the standard deviation of the pixel intensities through time. Positions with large differences in the pixel intensities through the stack appear brighter in this projection. Therefore, a black image highlights no variation between the frames over time (perfect drift correction), whereas signals highlight slight errors in the drift correction. For each *z*-slice, the standard deviation projection over time was generated and quantified using Fiji, and the results are shown as boxplots created by PlotsOfData (Postma and Goedhart, 2019), in which the boxes show the 25th and 75th percentiles, the whiskers represent the minimum and maximum values, and the median is marked with a line. No drift shows a high baseline value as specified noise was added during background homogenization. (D,E) The Pearson's correlation coefficient (PCC) (D) and peak signal-to-noise ratio (PSNR) (E) between the first and each subsequent frame were calculated. For PSNR, a higher value indicates a better drift correction. For Pearson's correlation coefficient, a value of 1 indicates perfect drift correction. For all panels, scale bars: 10 μm.

To assess the capabilities of Fast4DReg to correct 3D videos, we compared Fast4DReg results to two other state-of-the-art drift-correction methods available in Fiji (Schindelin et al., 2012): Correct3DD (Parslow et al., 2014) and Fijiyama (Fernandez and Moisy, 2021). For this purpose, two synthetic videos with known amounts of drift were created: one with no drift and another with a large amount of drift (Fig. 2A; Fig. S1A). As these videos were generated by duplicating an acquired single 3D stack and adding artificial drift, a perfect drift correction should generate near identical time frames as only the background noise will differ.

Visually, all three tools corrected the artificially drifting 3D videos regardless of the amount of drift (Fig. 2B; Movie 1). To carefully quantify the performance of these three software, we selected a *z*-slice and plotted the standard deviation projection of the corrected stack (Fig. 2C). Next, we calculated multiple image-similarity metrics between the first and each subsequent frame (Fig. 2D,E; Fig. S1B,C). Both assessment methods indicated that Fast4DReg performed better than Correct3DD or Fijiyama on our synthetic dataset (Movie 2; Fig. 2B–E; Fig. S1B,C). Importantly, these results demonstrated that using 2D intensity projections followed by 2D cross-correlation is a suitable method to correct drifting 3D videos.

### Fast4DReg is relatively resistant to noise

**Fig. 3.**
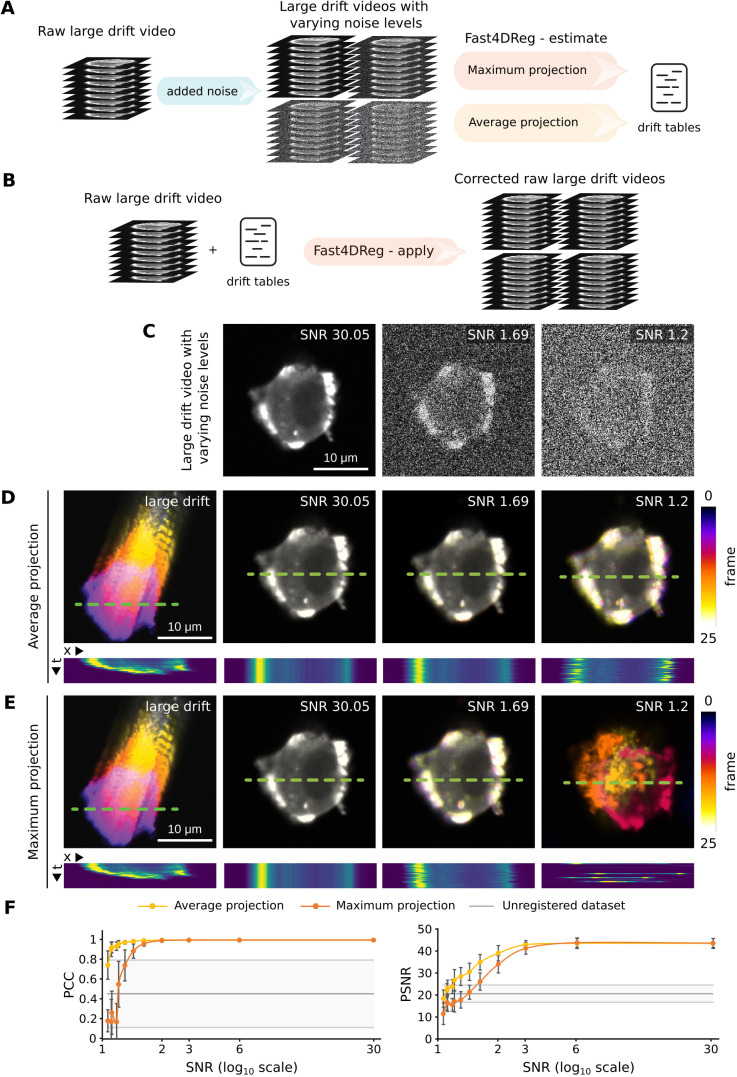
**Fast4DReg is relatively resistant to noise.** Twelve synthetic 3D video datasets with varying amounts of noise were created and corrected using Fast4DReg, either using maximum- or average-intensity projections. The drift tables were then applied to the original data to assess drift-correction accuracy. (A,B) Schematic illustrating the pipeline used to assess Fast4DReg sensitivity to noise. (C) Example of three noisy datasets used to assess Fast4DReg sensitivity to noise. (D,E) Fast4DReg drift-correction performance for three noisy datasets (C) was assessed using temporal color projections of a selected *z*-slice (middle of the cell) and kymographs (along the green dashed lines; dimensions, 25 μm × 25 frames). Note that Fast4DReg fails to register the images with an SNR of 1.2 when using maximum-intensity projections (E). (F) Fast4DReg drift-correction performance for the twelve noisy datasets was assessed using image-similarity metrics. The PSNR and PCC between the first and subsequent frames were calculated for each noise amount. For all panels, scale bars: 10 μm.

Live fluorescence imaging often requires using low illumination levels to avoid harming the sample, which can result in the acquisition of noisy images. In order to evaluate the sensitivity of Fast4DReg to noise, synthetic datasets with varying levels of noise were generated and processed using Fast4DReg. To assess and compare the results using image-similarity metrics, we applied the drift tables calculated by Fast4DReg to the original dataset, then calculated the image-similarity metrics on these corrected datasets (Fig. 3A,B). These analyses indicated that Fast4DReg was not affected by noise when the signal-to-noise ratio (SNR) was above 2. When the SNR was below 2, a decrease in performance could be observed. Interestingly, when the images started to be very noisy (SNR below 1.6), Fast4DReg performed much better when using average-intensity projections instead of maximum-intensity projections (Fig. 3C–F). Taken together, these results indicate that Fast4DReg is relatively resistant to noise and that, when correcting noisy data, it is more effective to use average-intensity projections.

### Fast4DReg is fast and successfully corrects drift from acquired 3D videos

Next, we assessed the suitability of Fast4DReg to correct drifts in acquired 3D biological images. We used a long 3D video of a human umbilical vein endothelial cell (HUVEC) monolayer labeled with silicon rhodamine (SiR)-actin and imaged using an Airyscan confocal microscope. We also registered this dataset with Correct3DD and Fijiyama. Although both Fast4DReg and Correct3DD produced good results when assessed visually (Movie 3), we failed to generate meaningful results with Fijiyama as the processing made the video drift even more than the raw data (data not shown).

**Fig. 4.**
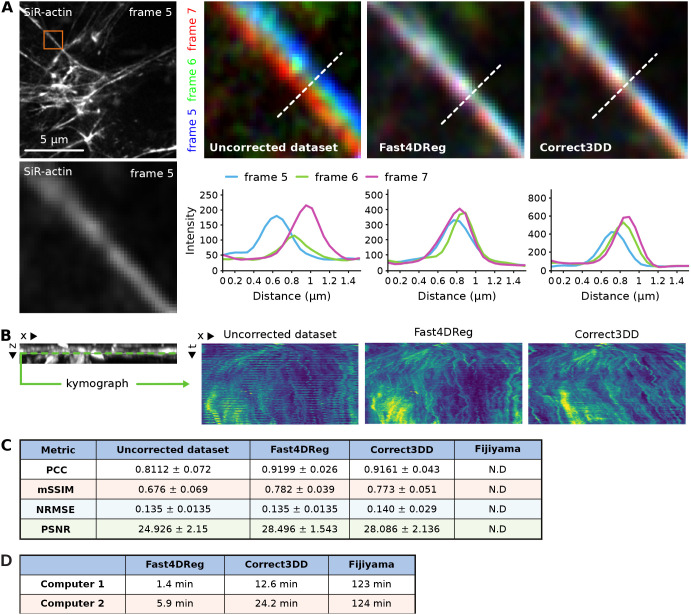
**Fast4DReg can rapidly correct axial and lateral drift in 3D videos.** A 3D video of HUVECs cells labeled with SiR-actin displaying a large *xyz*-drift was corrected with Fast4DReg and Correct3DD. (A) A region of interest containing a stress fiber that should remain immobile across multiple time points was chosen. Three consecutive frames were pseudo-colored blue, green and red, and merged. White indicates structural overlaps between the three frames. Line profiles along the dashed white lines to further study the overlap between frames were drawn as shown. (B) A kymograph (dimensions, 15 μm × 7146 s) of a selected line (monolayer ventral plane) in the *y*-projection was created to visualize the *z*-drift over time. (C) Image similarity metrics were calculated between each consecutive frame pair. Values show the mean±s.d. PCC, Pearson's correlation coefficient; mSSIM, mean structural similarity index; NRMSE, normalized root mean squared error; PSNR, peak signal-to-noise ratio. N.D., not determined. (D) Two computers, computer 1 (a high-performance desktop) and computer 2 (a laptop), were used to measure the speed of the correction methods. Shown values are the average times of three measurements.

To estimate the correction efficiency of Fast4DReg and Correct3DD on this dataset, we first searched for a structure that should remain immobile across multiple time points in the movie and chose a large stress fiber. We then color-coded three consecutive frames (one color per frame) and observed the overlaps of this stable structure between frames using line profiles (Fig. 4A). In the uncorrected movie, the stress fiber did not overlap in these three frames, clearly indicating drift. In the movies corrected by Fast4DReg and Correct3DD, the stress fiber overlap between frames improved, showing that the drift correction worked in both cases. Interestingly, the drift correction provided by Fast4DReg was superior here as the stress fiber overlap between the three frames was greater (Fig. 4A).

To visualize the axial drift-correction efficiency of Fast4DReg and Correct3DD on this dataset, we generated kymographs from the *y*-projections (Fig. 4B). In the original data, the kymograph showed a clear band pattern due to the microscope stage jumping cyclically. This banding pattern was improved in the movies corrected by Fast4DReg and Correct3DD. Still, it did not entirely disappear, indicating that although both registration methods worked well on this dataset, the correction was not perfect (Fig. 4B; Movie 3). This was perhaps because part of the data went out of the imaging volume several times. Of note, Correct3DD processing led to the monolayer slowly sinking over time, which is less desirable.

To obtain a quantitative estimate of the performance of Fast4DReg and Correct3DD on the HUVEC dataset, we measured the image-similarity metric between each adjacent frame pair for a selected *z*-plane in the corrected videos. Indeed, efficient drift correction should make successive frames more similar despite the inevitable biological changes. These biological changes would, however, lead to lower image-similarity metrics than those measured with our synthetic dataset (Figs 2 and 3), even if the data was perfectly registered. Using these metrics, we found that Fast4DReg and Correct3DD improved adjacent frame similarity on this dataset and performed similarly (Fig. 4C).

Finally, we assessed the computing time required by Fast4DReg, Correct3DD and Fijiyama to process the HUVEC dataset using two different computers. We found that Fast4DReg was four to nine times faster than Correct3DD and 20 to 90 times faster than Fijiyama when correcting the HUVEC dataset (Fig. 4D). These differences are relevant as the registration of datasets often requires tweaking hyperparameters to obtain the best possible results. Overall, Fast4DReg outperformed Correct3DD when correcting the HUVEC dataset, as the corrected data did not sink over time and the processing time was faster.

### Fast4DReg can register multichannel 3D videos

**Fig. 5.**
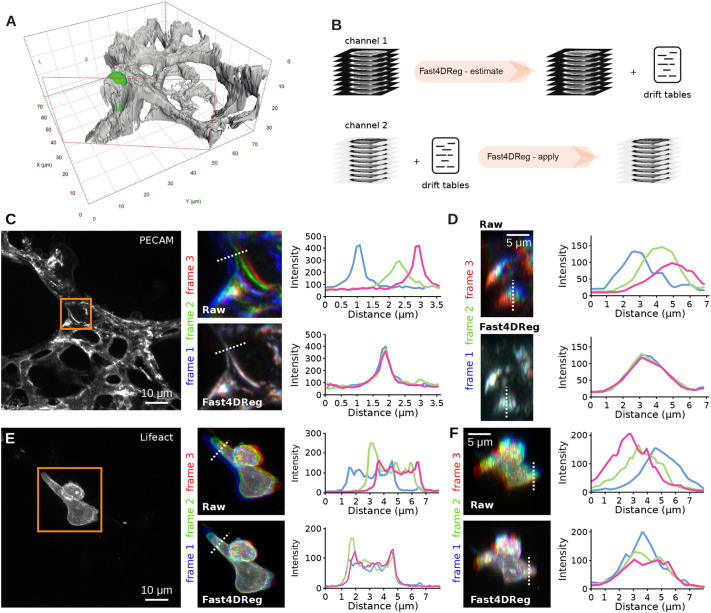
**Registration of a 3D multichannel video using Fast4DReg.** A 3D multichannel video of cancer cells migrating inside the lung vasculature was corrected using Fast4DReg. (A) 3D surface rendering of a selected time point (see also Movie 4) created using Arivis Vision4D. The lung vasculature is shown in grey, and the cancer cell (AsPC1) is in green. The red rectangle indicates the clipping plane used to render the image. (B) Schematic illustrating the pipeline used to correct a multichannel 3D video using Fast4DReg. For this dataset, the drift was first estimated using the vasculature images (channel 1), and the resulting drift table was then applied to the cancer cell images (channel 2). (C–E) Three consecutive frames of the vasculature (C) and cancer cell images (D) were pseudo-colored blue, green and red, and merged. White indicates structural overlaps between the three frames. Line profiles along the dotted white lines to further study the overlap between frames were drawn as shown. (C,E) *Z*-projections are displayed to visualize the lateral misalignments corrected by Fast4DReg. Scale bars: 10 μm. (D,F) *Y*-projections are displayed to visualize the axial misalignment corrected by Fast4DReg. Scale bars: 5 μm.

Next, we assessed the ability of Fast4DReg to correct acquired multichannel 3D videos. We used a movie of cancer cells migrating inside the lung vasculature, which was imaged *ex vivo* using an Airyscan confocal microscope (Fig. 5A; Movies 4 and 5). In this dataset, both the cancer cells and the vasculature were labeled, and a noticeable *xyz*-drift perturbated the visualization (Fig. 5; Movies 4 and 5). To correct these data with Fast4DReg, we first estimated the drift using the vasculature images. Once the drift was estimated, the same drift tables were then applied to the cancer cell images (Fig. 5B). Visually, Fast4DReg corrected this dataset very well (Movies 4 and 5). Using time projection of a selected *z*-plane and line-intensity profiles, we found that Fast4DReg could indeed successfully register the images of the vasculature (Fig. 5C). Importantly, applying the same drift tables also efficiently corrected the drift in the cancer cell images (Fig. 5D). We believe that the ability of Fast4DReg to apply drift tables to other datasets will significantly simplify the registration of multichannel data.

### Fast4DReg can also register misaligned 3D channel stacks

Finally, we tested whether Fast4DReg could be used to align 3D multichannel images instead of 3D videos. In this case, Fast4DReg uses the same pipeline as described for time series but first converts the channels into time frames.

**Fig. 6.**
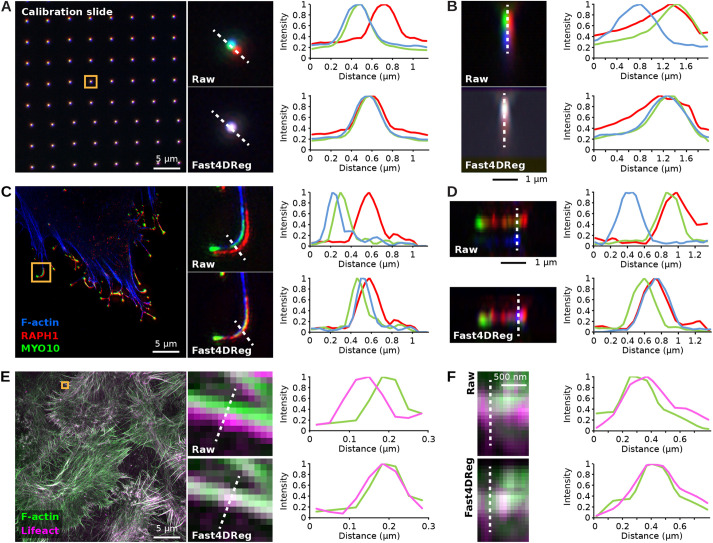
**Fast4DReg can align 3D multichannel images.** (A,B) A three-channel 3D calibration slide image was aligned using Fast4DReg. Merged images and line-intensity profiles (along the dashed white lines) are displayed to highlight the level of overlap between the three channels. (A) A *z*-projection is displayed to visualize the lateral misalignment corrected by Fast4DReg. (B) A *y*-projection of one of the calibration slide spots is displayed to illustrate the axial misalignment corrected by Fast4Dreg. (C,D) The drift table generated in A,B was then used to correct a 3D SIM image of a U2-OS cell expressing GFP-tagged lamellipodin (RAPH1, red) and MYO10–mScarlet (green), and labeled to visualize its actin cytoskeleton (blue). (C) A *z*-projection is displayed to visualize the lateral misalignment, evident in filopodia, corrected by Fast4Dreg. (D) A *y*-projection of one filopodium visualizes the axial misalignment corrected by Fast4Dreg. (E,F) A 3D SIM image of MCF10DCIS.com cells expressing RFP–Lifeact (magenta) and stained to visualize F-actin (green) was aligned using Fast4DReg directly. (E) A *z*-projection is displayed to visualize the lateral misalignment corrected by Fast4DReg. (F) A *y*-projection visualizes the slight axial misalignment corrected by Fast4DReg.

To test this approach, we registered, using Fast4DReg, a three-channel 3D image of a calibration slide. In this dataset, the raw images displayed significant *xyz*-misalignment owing to chromatic aberrations and the fact that the channels were acquired using different cameras (Fig. 6A,B). Using line-intensity profiles, we found that Fast4DReg could successfully register this dataset laterally and axially (Fig. 6A,B). Importantly, the drift tables measured using the calibration slide could then be used to correct any microscopy images acquired using the same conditions (Fig. 6C,D). Combined with the Fast4DReg batch-processing mode, we envision that the indirect channel alignment approach described here will be advantageous when performing colocalization analyses.

However, not all datasets have a corresponding calibration slide or bead image, so we also tested the ability of Fast4DReg to correct misaligned channels directly. In this case, we used a two-channel 3D image that was acquired to train a supervised image-restoration deep-learning algorithm (von Chamier et al., 2021; Weigert et al., 2018). As with the previous example, Fast4DReg performed well in registering this dataset (Fig. 6E,F). It is worth noting that a direct channel alignment approach might only work for some datasets, as Fast4DReg requires structural overlap between the channels to perform the registration. Additionally, we do not recommend directly aligning images aimed for colocalization analysis as it might lead to artefactual results.

## DISCUSSION

In live microscopy, sample drifting can be a significant challenge, and implementing post-processing drift-correction pipelines is not always fast or straightforward. Here, we developed Fast4DReg, an ImageJ or Fiji-based tool that can quickly correct axial and lateral drift in 2D and 3D videos. We show that Fast4DReg can outperform two open-source 3D drift-correction tools on our test datasets. A significant advantage of Fast4DReg is that it can correct 3D videos in a fraction of the time compared to other tested tools and comes with an easy-to-use graphical user interface (Fig. S2). Fast4DReg speed is likely due to two factors: (1) using 2D projections greatly simplifies the computations required and (2) using CPU multithreading further accelerates the 2D cross-correlation process. In the future, Fast4DReg speed could be further improved by enabling graphic card acceleration.

Despite its performance, Fast4DReg has several limitations. Firstly, Fast4DReg can only perform translations when correcting a dataset. Rotation, scaling or shearing transformations are not supported, although these should not be required to correct most time-course video or multichannel microscopy datasets. Secondly, the channel alignment is limited to images with structural conservation between channels or requires calibration slides or beads images to compute the shift maps.

With Fast4DReg, we demonstrate that using intensity projections followed by 2D cross-correlation is a quick and efficient way to register various multidimensional data types, including 3D videos and 3D multichannel datasets. In the future, it would be interesting to assess the suitability of using 3D cross-correlation directly to register similar images. But using 3D cross-correlation will likely impede processing times.

To promote adoption by the community, Fast4DReg is available through a Fiji update site, GitHub and Zenodo. We also provide test datasets and detailed step-by-step instructions. With Fast4DReg, we hope to make the process of multidimensional data registration more straightforward and faster, and, therefore, more accessible to the community.

## MATERIALS AND METHODS

### Algorithms

To estimate the lateral or axial drift of a 3D video, Fast4DReg creates *z-* or *y*-intensity projections for each time point to create a 2D video. Fast4DReg then estimates the linear drift between the reference and moving frames by calculating their CCM. In Fast4DReg, in a similar fashion to the work of Sun (2002), the cross-correlation between two images is calculated by performing a discrete Hartley transform on both images, followed by a multiplication of one of the transformed images by the complex conjugate of the other. The result of this multiplication is then inversely transformed back to real space, generating the CCM. A bicubic spline interpolation is then used to upscale the CCM and achieve subpixel precision. The upscaled CCM is normalized by calculating the Pearson's correlation coefficient between the two images shifted according to the minimum and maximum values of the upscaled CCM. Finally, the linear shift between the two images is then calculated by taking the global maximum peak of the normalized up-scaled CCM (as demonstrated by Laine et al., 2019).

Fast4DReg can also be used to register channels from misaligned 3D stacks. In this case, Fast4DReg simply converts the channels into time frames before applying the Fast4DReg drift-correction pipeline described above. As a note of caution, cross-correlation only works well to register channels in which similar structures or cells are labeled. Importantly Fast4DReg can also register 2D video and 2D multichannel images either one at a time or in batches.

Fast4DReg can run on any computer on which Fiji (Schindelin et al., 2012) and the Bio-Formats (Linkert et al., 2010) plugin are installed. Fast4DReg also has a memory-saving mode that allows the registration of larger datasets using a computer with limited resources (processing time available in Fig. S1D).

Fast4DReg expects as input one or multiple single-channel 2D or 3D videos. Fast4DReg outputs corrected files, drift tables, drift plots and a settings file. Owing to Bio-Formats (Linkert et al., 2010), Fast4DReg can handle various image formats as input. Fast4DReg can be tested using our test datasets available on Zenodo (https://zenodo.org/record/7514913). Fast4DReg is written using a combination of an ImageJ macro and Java, and is distributed via an ImageJ update site. The installation procedure and up-to-date, step-by-step instructions are available on the Fast4DReg GitHub page (https://github.com/guijacquemet/Fast4DReg).

### Cells

AsPC1 cells (a pancreatic ductal adenocarcinoma cell line) were purchased from American Type Culture Collection (CRL-1682) and grown in Roswell Park Memorial Institute medium (Thermo Fisher Scientific, 11875093) supplemented with 10% fetal bovine serum (FBS) (Biowest, S1860). HUVECs were purchased from PromoCell (C-12203) and grown in endothelial cell growth medium (PromoCell, C-22010). U2-OS osteosarcoma cells were purchased from the Leibniz Institute DSMZ, German Collection of Microorganisms and Cell Cultures (Braunschweig, Germany; ACC 785) and grown in Dulbecco's modified Eagle medium (DMEM; Merck, D5671) supplemented with 10% FBS. MCF10DCIS.com Lifeact–RFP cells were generated previously (Jacquemet et al., 2017) and cultured in a 1:1 mix of DMEM and F12 (Merck, 51651C) supplemented with 5% horse serum (Gibco, 16050122), 20 ng/ml human epidermal growth factor (Merck, E9644), 0.5 mg/ml hydrocortisone (Merck, H0888-1G), 100 ng/ml cholera toxin (Merck, C8052-1MG), 10 mg/ml insulin (Merck, I9278-5ML) and 1% (vol/vol) penicillin/streptomycin (Merck, P0781-100ML). All cell lines tested negative for mycoplasma. AsPC1 cells were authenticated by DSMZ. All other cell lines were not authenticated.

### Datasets with synthetic drift

The synthetic drift datasets were created by duplicating a 3D stack image 25 times and artificially adding a known amount of *x-*, *y*- and *z*-drift between each frame. The original image was that of an AsPC1 cell expressing Lifeact–mScarletI migrating inside the vasculature of a zebrafish embryo. This image was acquired using a 3i CSU-W1 spinning-disk confocal microscope equipped with a 40× water immersion objective (NA 1.15) and a Hamamatsu sCMOS Orca Flash camera. The microscope was controlled using the Slidebook 6 software (Intelligent Imaging Innovations, Inc.; https://www.intelligent-imaging.com/slidebook).

The amount of drift added corresponds to the drift typically observed in our live-cell imaging experiments. Using this method, two videos were created: one with no drift (ground-truth video) and one with a large drift (across the field of view) (see also Fig. S1A). After the drift was simulated, the image background was made homogeneous via pixel intensity subtraction and by adding specified noise using Fiji (‘add specified noise’ function). The *xy-* and *z*-drift in these synthetic datasets was corrected using Fast4DReg, Correct3DD and Fijiyama. For each software, the parameters providing the best possible drift correction were chosen (the settings used are described in Table S1).

After correcting the drift in the synthetic datasets, the images were first cropped to be the same size (352×275 pixels, 69 *z*-slices, 25 frames) using Fiji. The drift-correction performance was then quantified by measuring image-similarity metrics between frames (the reference frame was the first frame) of a selected z-slice (*z*-slice 51) using a custom-made Jupyter notebook (available in Zenodo). This *z*-slice was selected as it was in the middle of the cell.

### The noisy synthetic drift dataset

To generate the 12 noisy synthetic drift datasets, a specified amount of Gaussian noise was added to the original synthetically drifting dataset using Fiji (‘add specified noise’ function). The added selected Gaussian noise had standard deviations of 0, 5000, 10,000, 15,000, 20,000, 25,000, 30,000, 35,000, 40,000, 45,000, 50,000 and 60,000, yielding images with SNR of 30.053, 5.586, 2.964, 2.111, 1.686, 1.478, 1.327, 1.227, 1.196, 1.127, 1.119 and 1.070, respectively. The SNR was calculated by dividing the mean cell signal by the mean background signal. All 12 generated noisy synthetic drifts were corrected using Fast4DReg using maximum- or average-intensity projections (the settings used are described in Table S1). The generated drift tables were then used to correct the original large drift dataset (Fig. 3A,B). Corrected images were then cropped to be the same size (192×192 pixels, 69 *z*-slices, 25 frames) using Fiji. The drift-correction performance was then quantified by measuring image-similarity metrics between frames (the reference frame was the first frame) of a selected *z*-slice (z-slice 51) using a custom-made Jupyter notebook (available in Zenodo). This *z*-slice was selected as it was in the middle of the cell.

### The HUVEC monolayer dataset

The HUVEC monolayer dataset consists of a 3D video of HUVECs labeled with SiR-actin (Spirochrome). The video was acquired using a laser scanning confocal LSM880 microscope (Zeiss) equipped with an Airyscan detector (Carl Zeiss) and a 63× oil (NA 1.4) objective. The microscope was controlled using Zen Black (2.3) (Zeiss), and the Airyscan detector was used in standard super-resolution mode. This dataset has 200 frames (488×488 pixels) and 24 *z*-slices. This dataset was corrected using Fast4DReg, Correct3DD and Fijiyama using the parameters providing the best possible drift correction (the settings used are described in Table S1). The correction performance was quantified by measuring image-similarity metrics between adjacent frames (the reference frame was the previous frame) of a selected *z*-slice (*z*-slice 8) using a custom-made Jupyter notebook.

Two computers were used to compare the execution times of all compared methods: computer 1 (operating system, Windows; processor, AMD Ryzen 7 5800X 8-Core; graphics card, GeForce GTX 3080; RAM, 32 GB; Fiji version 1.53q) and computer 2 [operating system, macOS; processor, M1 chip (8-core CPU, 8-core GPU); RAM: 16 GB; Fiji version 1.53q].

### The mouse lung dataset

The mouse lung dataset (624×626 pixels, 55 *z*-slices, eight frames, two channels) consists of a 3D video of an AsPC1 cell expressing Lifeact–mNeonGreen migrating inside the lung vasculature. Briefly, labeled AsPC1 cells were injected into the tail vein of a 10-week-old immunocompromised female mouse (Hsd: Athymic Nude-*Foxn1^nu^* strain). The mouse was euthanized shortly after injection, and precision-cut lung slices were prepared (Puttur et al., 2019). The National Animal Experiment Board authorized all animal studies, and per The Finnish Act on Animal Experimentation (animal license number 12558/2021). The lung endothelial cells were labeled using an Alexa Fluor 488-conjugated anti-PECAM antibody (1:100, BioLegend, 102413). The precision-cut lung slices were then imaged using an Airyscan confocal LSM880 microscope (Carl Zeiss) equipped with a 63× water (NA 1.15) objective. The *xy*- and *z*-drift in this dataset was corrected with Fast4DReg using the PECAM staining (settings used described in Table S1). The drift table generated by Fast4DReg was then applied to the second channel (cancer cells), after which these channels were merged using Fiji. Line-intensity profiles of three consecutive frames of selected structures were measured using Fiji to quantify the correction. Arivis Vision4D (v 3.5.0, Zeiss, https://www.zeiss.com/microscopy/en/products/software/arivis-vision4d.html) was used for the 3D reconstruction of the time-lapse movies. Surface rendering was performed using the 'Extract Isosurface' function.

### The calibration slide dataset

The calibration slide dataset (1024×1024 pixels, 25 *z*-slices, three channels) was created by imaging a channel calibration slide (Argolight HM) using a DeltaVision OMX v4 (GE Healthcare Life Sciences) microscope fitted with a 60× Plan-Apochromat objective lens, 1.42 NA (immersion oil refractive index of 1.516), used in wide-field illumination mode. The emitted light was collected on a front-illuminated pco.edge sCMOS camera (pixel size 6.5 mm, readout speed 95 MHz; PCO) controlled by SoftWorx (AppliedPrecision). The *xy*- and *z*-drift in this dataset was corrected using Fast4DReg (the settings used are described in Table S1).

### The filopodia dataset

The filopodia dataset (1024×1024 pixels, 17 *z*-slices, three channels) consists of a 3D structured illumination microscopy (SIM) image of a U2-OS cell expressing GFP-tagged lamellipodin and MYO10–mScarlet, and labelled with SiR-actin (Popovic et al., 2023). This dataset was acquired using a DeltaVision OMX v4 (GE Healthcare Life Sciences) microscope fitted with a 60× Plan-Apochromat objective lens, 1.42 NA (immersion oil refractive index of 1.516) used in SIM illumination mode (five phases and three rotations). The emitted light was collected on a front-illuminated pco.edge sCMOS camera (pixel size 6.5 mm, readout speed 95 MHz; PCO) controlled by SoftWorx. The *xy*- and *z*-drift in this dataset was corrected using Fast4DReg using the drift table computed using the calibration slide dataset (the settings used are described in Table S1).

### The DCIS.com filopodia dataset

The DCIS.com Filopodia dataset consists of a SIM image of MCF10DCIS.COM Lifeact–RFP cells labeled with phalloidin. Briefly, MCF10DCIS.COM Lifeact–RFP cells (Jacquemet et al., 2017) were grown on high-tolerance glass-bottomed dishes (MatTek Corporation, coverslip 1.5). Cells were fixed and permeabilized simultaneously using 4% (wt/vol) paraformaldehyde and 0.25% (vol/vol) Triton X-100 for 10 min. Cells were then washed with PBS, quenched using a solution of 1 M glycine for 30 min, and incubated with Alexa Fluor 488 phalloidin (1:200 in PBS; A12379, Thermo Fisher Scientific) at 4°C overnight until imaging. Samples were washed three times in PBS, mounted in Vectashield (Vectorlabs), and imaged using a DeltaVision OMX v4 (GE Healthcare Life Sciences) used in SIM illumination mode (five phases and three rotations). The microscope was fitted with a ×60 Plan-Apochromat objective lens (1.42 NA, immersion oil refractive index of 1.516). The fluorescent light was collected using front-illuminated pco.edge sCMOS camera (pixel size 6.5 μm, readout speed 95 MHz; PCO). The high SNR ratio images were acquired from the phalloidin-488 staining using acquisition parameters optimal to obtain high-quality SIM images (50 ms of exposure time, 10% laser power). The low SNR ratio images were acquired from the Lifeact-RFP channel using acquisition parameters more suitable for live-cell imaging (100 ms of exposure time, 1% laser power). The *xy*- and *z*-drift in this dataset was corrected using Fast4DReg (the settings used are described in Table S1).

### Metrics

To quantitatively assess the drift-correction performance of Fast4DReg and the other tools tested, four image-similarity metrics were used. These metrics were calculated using a custom-made Jupyter notebook (modified from Laine et al., 2021). This notebook is available on Zenodo (https://zenodo.org/record/7514913).

Pearson's correlation coefficient (PCC) measures the linear correlation between two images. A PCC value of 1 indicates a perfect linear relationship, or perfect similarity, between the two images. The mean structural similarity index (mSSIM) evaluates the similarity of two images based on their contrast, luminance and structural content. An mSSIM value of 1 indicates that the two images are perfectly similar. The peak SNR ratio (PSNR) is a metric that compares the peak signal amplitudes of two images and is typically expressed in decibels. A higher PSNR value indicates greater similarity between the two images. The normalized root mean squared error (NRMSE) measures the average difference between the pixel intensity in two images. A lower NRMSE value indicates greater similarity between the images.

### Fast4DReg downloads and source code

Fast4DReg, the generator of synthetic drift, and the notebook used to make the image-similarity measurements (all under MIT licenses) are available on GitHub (https://github.com/guijacquemet/Fast4DReg) and their source code is archived on Zenodo (https://zenodo.org/record/7514913). Fast4DReg is also available through a Fiji update site (https://imagej.net/plugins/fast4dreg).

## Supplementary Material

10.1242/joces.260728_sup1 Supplementary information
