# Supplementary material for: Fast4DReg – fast registration of 4D microscopy datasets
Source: J Cell Sci. 2023 Feb 27;136(4):jcs260728. doi: 10.1242/jcs.260728 (PMC10022679; doi:10.1242/jcs.260728)
Supplement: Supplementary information [file joces-136-260728-s1.pdf]

**A**

Large drift (pixels)

| Frame | X-drift | Y-drift | Z-drift |
|---|---|---|---|
| 1 | -0.1846 | -0.842 | 0.7736 |
| 2 | 1.1534 | 0.69 | -1.0418 |
| 3 | 0.3099 | 1.261 | -0.5265 |
| 4 | 1.3837 | 1.961 | -2.517 |
| 5 | 2.8785 | 1 | -3.3751 |
| 6 | 3.221 | -0.072 | -4.0643 |
| 7 | 4.83 | -2.012 | -2.4789 |
| 8 | 6.2691 | -4.293 | -4.9687 |
| 9 | 7.1845 | -7.21 | -5.6736 |
| 10 | 8.8191 | -11.375 | -7.2127 |
| 11 | 12.1096 | -13.948 | -6.6842 |
| 12 | 13.6142 | -18.579 | -7.6793 |
| 13 | 15.5335 | -25.244 | -7.4236 |
| 14 | 18.8507 | -32.933 | -7.7894 |
| 15 | 21.5718 | -37.042 | -9.3144 |
| 16 | 25.3066 | -45.681 | -8.9694 |
| 17 | 28.4077 | -52.989 | -9.1761 |
| 18 | 32.8398 | -60.376 | -9.9103 |
| 19 | 34.7591 | -71.216 | -10.8287 |
| 20 | 40.0535 | -79.337 | -10.9413 |
| 21 | 42.8704 | -90.376 | -11.5039 |
| 22 | 47.4757 | -100.918 | -13.4116 |
| 23 | 51.8525 | -112.391 | -14.2919 |
| 24 | 56.8755 | -124.903 | -14.3824 |
| 25 | 62.7437 | -137.609 | -14.8706 |

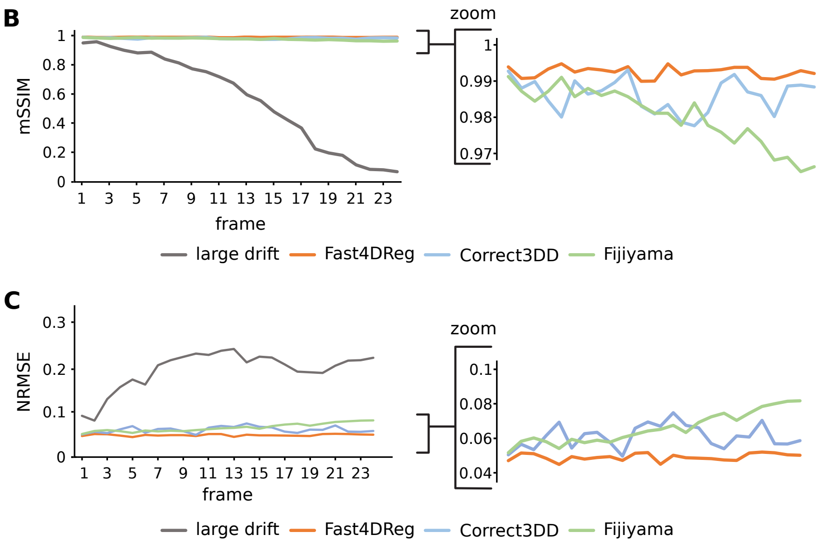


**D**

| | Computer 1 | Computer 2 |
|---|---|---|
| **Fast4DReg** | 1.4 min | 5.9 min |
| **Fast4DReg (RAM saving mode)** | 1.15 min | 4.5 min |

**Fig. S1. Fast4DReg outperforms Correct3DD and Fijiyama on a synthetic 3D + t dataset.** (**A-C**) Two synthetic 3D video datasets were created, one with no drift, and another with a large amount of drift (see also Fig. 2). (**A**) Drift table indicating the amount of drift added to create the large drift dataset. (**B and C**) The large drift dataset was then corrected using Fast4DReg, Correct3DD, and Fijiyama, and the registration quality assed using image similarity metrics. The mSSIM (**B**) and NRMSE metrics (**C**) between the first and each subsequent frame were calculated. For mSSIM a value of 1 indicates perfect drift correction, for NRMSE, a lower value indicates a better drift correction. (**D**) Comparison of Fast4DReg processing time when the RAM saving mode is enabled (HUVEC monolayer -dataset).

Journal of Cell Science • Supplementary information

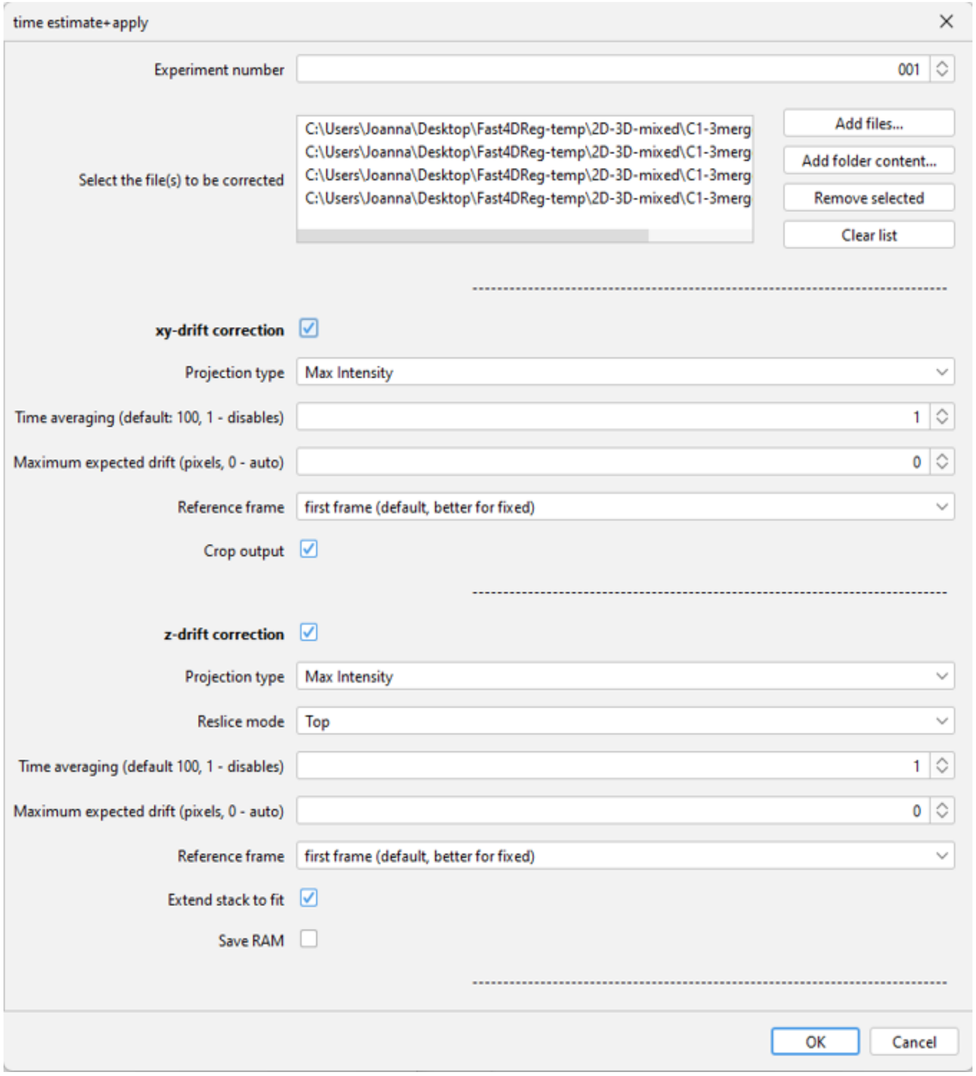


**Fig. S2. Fast4DReg user interface.** Screenshot highlighting Fast4DReg user interface.

**Table S1.** Table S1 lists the Fast4DReg, Corect3DD, and Fijiyama settings used to analyze the various datasets presented in this study.

Click here to download Table S1

Journal of Cell Science • Supplementary information

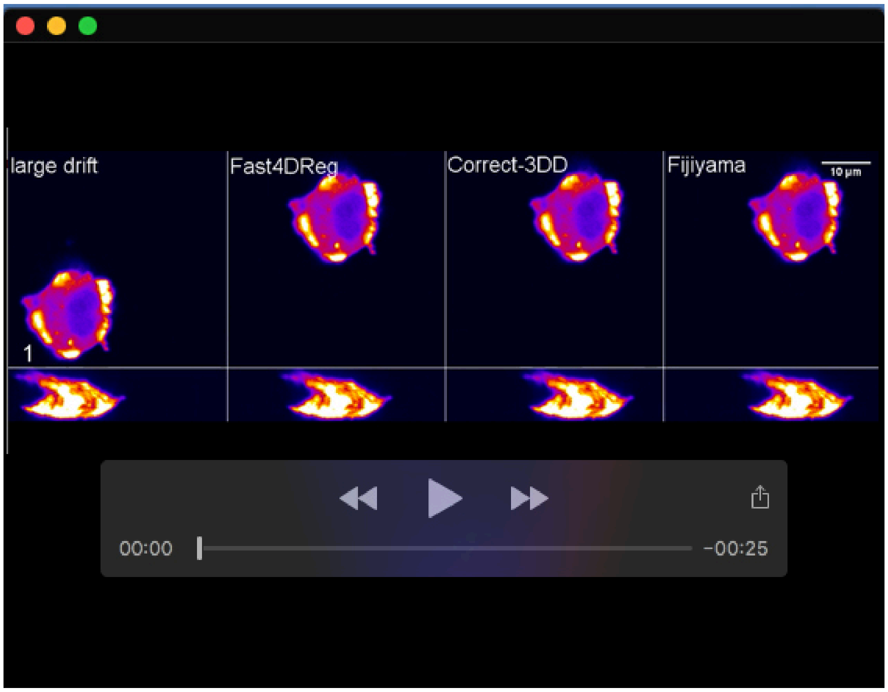


**Movie 1. Dataset with a large amount of drift corrected using Fast4DReg, Correct3DD, and Fijiyama.** The top panels show a selected z-plane of the uncorrected and corrected videos. To visualize the axial drift, the bottom panels show the y projection of the uncorrected and corrected videos. Note that the drift correction tools generate a cropped image, causing a mismatch in the location of the cell compared to the raw data. Scale bar 10 µm.

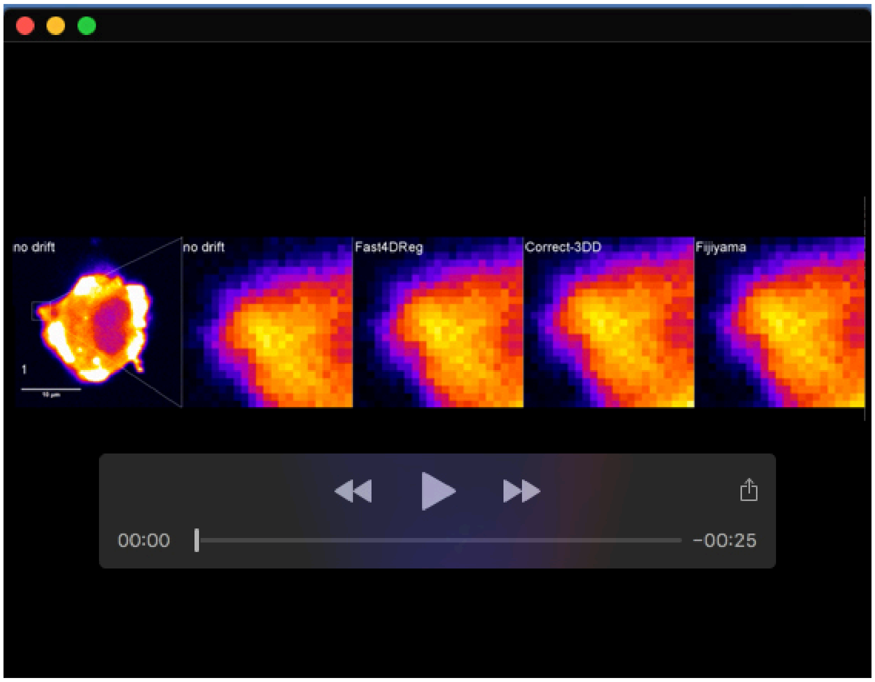


**Movie 2. Comparison of the drift correction performance of Fast4DReg Correct3DD and Fijiyama.** A selected region of ground truth and drift-corrected videos was magnified to visualize the drift-correction performance of Fast4DReg, Correct3DD, and Fijiyama. Scale bar 10 µm.

Journal of Cell Science • Supplementary information

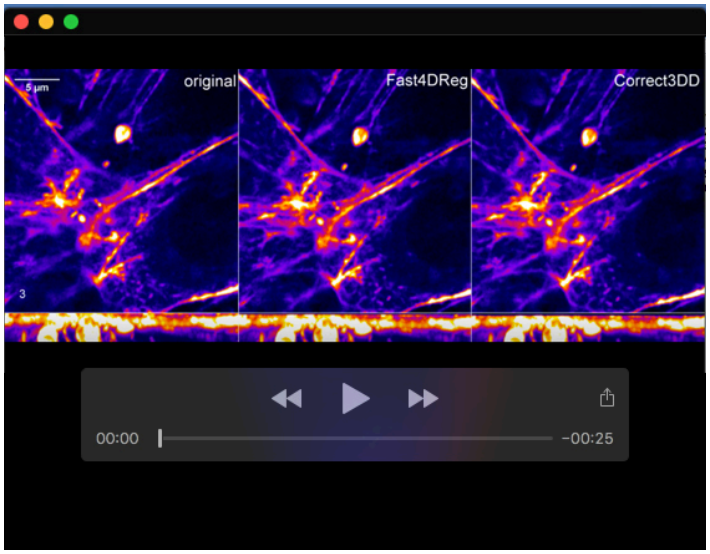


**Movie 3. HUVEC-monolayer dataset corrected using Fast4DReg and Correct3DD.** HUVEC cells labeled with SiR-actin were imaged in 3D using a Zeiss 880 Airyscan confocal microscope. This dataset suffered from significant drift, corrected using Fast4DReg and Correct3DD. The top panels show a selected z-plane of the uncorrected and corrected videos. The bottom panels show the y-projection of the uncorrected and corrected videos to visualize the axial drift. Scale bar 5 µm.

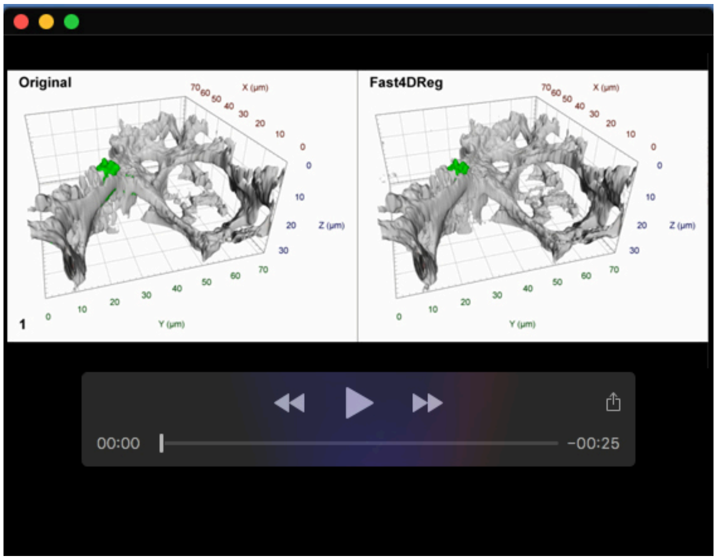


**Movie 4. 3D surface rendering of the mouse lung dataset.** AsPC1 cancer cell migrating inside the mouse lung vasculature was imaged ex-vivo in 3D using a Zeiss 880 Airyscan confocal microscope. The original dataset suffered from lateral and axial drift, which was corrected using Fast4DReg. 3D surface renderings of the original and Fast4DReg corrected 3D videos were created using Arivis Vision4D.

Journal of Cell Science • Supplementary information

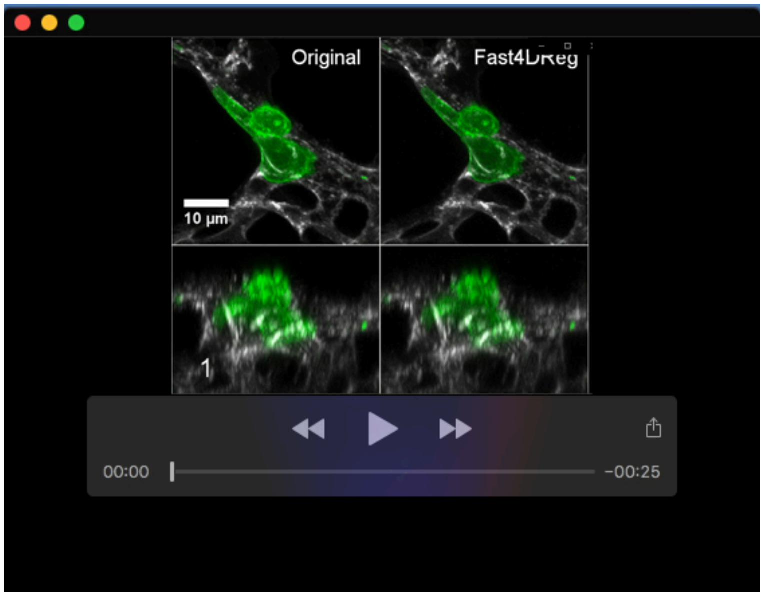


**Movie 5. The mouse lung dataset corrected using Fast4DReg.** AsPC1 cancer cell migrating inside the mouse lung vasculature was imaged ex-vivo in 3D using a Zeiss 880 Airyscan confocal microscope. The original dataset suffered from lateral and axial drift, which was corrected using Fast4DReg. The top panels show z-projection of the uncorrected and corrected videos. The bottom panels show the y-projection of the uncorrected and corrected videos to visualize the axial drift. Scale bar 10 µm.

Journal of Cell Science • Supplementary information
