# Supplementary material for: Fast4DReg – fast registration of 4D microscopy datasets
Source: J Cell Sci. 2023 Feb 27;136(4):jcs260728. doi: 10.1242/jcs.260728 (PMC10022679; doi:10.1242/jcs.260728)
Supplement: Supplementary file 1 [file joces-136-260728_review_history.pdf]

Fast4DReg - fast registration of 4D microscopy datasets

Joanna W. Pylvänäinen, Romain F. Laine, Bruno M. S. Saraiva, Sujan Ghimire, Gautier Follain, Ricardo Henriques and Guillaume Jacquemet
DOI: 10.1242/jcs.260728

Editor: Michael Way

Review timeline

Original submission:	16 October 2022
Editorial decision:	11 November 2022
First revision received:	13 January 2023
Editorial decision:	23 January 2023
Second revision received:	24 January 2023
Accepted:	25 January 2023

Original submission

First decision letter

MS ID#: JOCES/2022/260728

MS TITLE: Fast4DReg: Fast registration of 4D microscopy datasets

AUTHORS: Joanna Pylvänäinen, Romain F Laine, Bruno MS Saraiva, Sujan Ghimire, Gautier Follain, Ricardo Henriques, and Guillaume Jacquemet
ARTICLE TYPE: Tools and Resources

We have now reached a decision on the above manuscript.

To see the reviewers' reports and a copy of this decision letter, please go to: https://submit-jcs.biologists.org and click on the 'Manuscripts with Decisions' queue in the Author Area. (Corresponding author only has access to reviews.)

As you will see, the two reviewers raise a number of substantial criticisms that prevent me from accepting the paper at this stage. They suggest, however, that a revised version might prove acceptable, if you can address their concerns all of which I think will improve and strengthen your manuscript. If you think that you can deal satisfactorily with the criticisms on revision, I would be pleased to see a revised manuscript.

Please ensure that you clearly highlight all changes made in the revised manuscript. Please avoid using 'Tracked changes' in Word files as these are lost in PDF conversion.

I should be grateful if you would also provide a point-by-point response detailing how you have dealt with the points raised by the reviewers in the 'Response to Reviewers' box. Please attend to all of the reviewers' comments. If you do not agree with any of their criticisms or suggestions please explain clearly why this is so.

Reviewer 1

Advance summary and potential significance to field

© 2023. Published by The Company of Biologists under the terms of the Creative Commons Attribution License (https://creativecommons.org/licenses/by/4.0/).

The authors present Fast4DReg, a collection of scripts for the popular FIJI platform for the correction of axial and lateral drift in time-lapse imaging datasets. Fast4DReg can also be used to correct misalignment between channels in multi-channel datasets. The principles underlying the software's operation are illustrated with some nice schematics and the authors use a variety of test datasets to test the performance of their software against other state-of-the-art tools. While the performance of Fast4DReg appears to be comparable to another freely-available tool (Correct 3DD), the results presented suggest that Fast4DReg is significantly faster, which would undoubtedly make Fast4DReg of interest to the broader community. However, there are some shortcomings associated with the demonstration of the software that should be addressed to strengthen the case for the software's adoption.

Comments for the author

Firstly, while captured as 3D image stacks, the test datasets used are largely two-dimensional in nature. For example, the registration slide dataset is a two-dimensional array of dots. It would be useful to know how Fast4DReg performs on truly 3D data and how it compares to other software on such datasets.

Secondly, there is a lack of detail concerning the algorithmic underpinnings of Fast4DReg. More details on how the cross-correlation matrices are calculated is warranted, given how central this is to the method. For example, what correlation coefficient is used? And how is the peak determined? How does Fast4DReg discriminate between multiple peaks in the CCM?

Finally, in quantifying the registration accuracy of Fast4DReg relative to other tools, the authors rely solely on Pearson's Correlation Coefficient. Is this the same correlation coefficient used to construct the CCMs? If so, it is perhaps not surprising that Fast4DReg performs so well! How do the various tools compare when registration is evaluated using other metrics?

Other General Points
* I have reservations about "advertising" this tool as a means to correct misalignment between channels. This could lead to the artificial localisation of signals that are not in fact co-localised at all. The authors should stress that this functionality should only be used for co-labelled structures (such as fluorescent beads or calibration slides). A note of caution is sounded, but I think this needs to be expanded upon. Using a calibration slide should always be the recommended approach for correcting channel misalignment.
* How do the authors suggest users differentiate between drift and sample migration?
* How would it handle very large datasets? It is stated that "Fast4DReg has a RAM-saving mode that allows the registration of large datasets" (and "large 3D videos" are referenced in the abstract), but none of the test datasets are particularly large. It is also stated that this RAM-saving mode makes processing times "slightly longer", but this is not quantified - this is an important omission, given how processing speed is one of the main advantages of Fast4DReg.
* Correlation Coefficients (particularly Pearson's) are sensitive to noise - how well does Fast4DReg function on datasets with a low signal-to-noise ratio?

Specific Points:

Introduction
* With regard to the opening paragraph, how prevalent is this problem of drift? If the microscope and sample are properly equilibrated, drift due to temperature change should be minimal, especially if the microscope in question is equipped with hardware-based drift-correction tools (such as Nikon's PFS).
* "Multiview Reconstruction, which was designed to register large lightsheet fluorescence microscopy datasets, uses beads and/or segmented structures in the imaging volume to perform the registration" - this is not true. Registration can be accomplished in the absence of landmarks. Also worth noting that this registration is performed in 3D, albeit (typically) with downsampled images.

Implementation, operation, and test datasets
* What is the origin of the original image in Dataset 1?
* It seems a bit odd to include the computer specifications in their current location (under Dataset 2) - do these specifications only apply to Dataset 2?

© 2023. Published by The Company of Biologists under the terms of the Creative Commons Attribution License (https://creativecommons.org/licenses/by/4.0/).

* "The Filopodia-dataset (1024x1024 px, 17 z-slices, 3 channels) consists of a 3D structured illumination microscopy (SIM) image of a U2OS cell expressing a GFP-tagged Lamellipodin fragment, MYO10-mScarlet, and labeled to visualize its actin cytoskeleton using sirActin" - this is worded confusingly. I'm not completely sure what cell structures are labelled with what fluorophores.
* Dataset 2: the drift in this dataset looks very odd and periodic in nature? Could the authors explain the source of this effect?
* Dataset 4: similar to the above, the channel misalignment here seems excessive?

Use Cases
* "To carefully quantify the performance of these three software, we selected a z-slice..." - how? And why?
* In Fig 2C, is this a projection over time? Based on the figure legend, this would appear to be the case, but the description is confusing. For example, "This projection takes the standard deviation of the pixel intensities through the stack" suggests that we are looking at a Z-projection.
* In Fig 2D, the standard deviation is calculated on a slice-by-slice basis - why? Why not calculate the SD over the entire volume through time at once?
* The differences shown in Fig 2F are tiny - I don't think Pearson's is the best metric to use here, as the images contain large areas of background (which is undoubtedly skewing the correlation coefficients upwards). The contrast with Fig 3c is stark, although the two datasets are obviously quite different in nature (one is time-lapse, the other is not)
* I'm not sure I understand the point of Fig 3A? It seems highly selective (spatially and temporally) and is made somewhat redundant by subsequent panels
* Similarly, Fig 3B is confusing - it's pretty difficult to tell if Fast4DReg is better or worse (or comparable?) than Correct 3DD based on these kymographs. Also, how was the location in Z selected for the kymograph? Is it the mid-point of the stack?
* "In the original data, the kymograph shows a clear band pattern due to the microscope stage jumping cyclically." - why is the microscope stage doing this?!?
* "Indeed, we consider that efficient drift correction will make successive frames more similar to one another despite the inevitable biological changes." - this is obviously a problem that the authors should elaborate on
* "Using this metric, we find that Fast4DReg performed equally or slightly better than Correct 3DD drift on this dataset" - this statement should be more quantitative. What does "equally" or "slightly better" mean?
* "We chose images of cells displaying filopodia as these actin structures are very thin and narrow, rendering them almost two-dimensional" - what happens if such structures are not available? The authors touch on this problem by stating that "This approach will not work with all images, as the registration method used here will work only if sufficient structural overlap exists between the channels", but then go on to state that they "envision that the approach described here can be beneficial in correcting channel misalignment when no calibration data is available". As stated above, using a calibration slide should always be the recommended approach for correcting channel misalignment to avoid apparent co-localisation of signals that are not, in reality, co-localised.

<u>Reviewer 2</u>

Advance summary and potential significance to field

I had the pleasure to review a manuscript titled "Fast4DReg: Fast registration of 4D microscopy datasets" by Joanna W. Pylvänäinen et al. The manuscript describes a 3D/4D image registration tool tailored to correct drift in microscopy timelapse imaging data. The presented problem is highly relevant and the demonstrated solution is a fantastic achievement - in particular in the context of processing speed and user-friendlyness. The manuscript is written in excellent english and has a clear red line. The Figures are outstandingly well prepared and the authors successfully explain a highly complex topic in an accessible fashion. I am fully convinced that the authors give a new powerful tool into the hands of end-users. Practically speaking, Fast4DReg makes a functionality available to ImageJ/Fiji users that was not available yet and highly demanded. The most commonly used tool for similar purposes (StackReg) is available for 2D images only and lacks substantial features the authors implemented in Fast4DReg. In this context, I would like to highlight a sentence from the pipeline section: "Notably, the drift table can then be applied to correct other images using the same parameters" This feature is super amazing and presumably response to user

© 2023. Published by The Company of Biologists under the terms of the Creative Commons Attribution License (https://creativecommons.org/licenses/by/4.0/).

requests. I have heard StackReg users a thousand times complaining that exactly this feature is not available. Btw. StackReg could be cited as well as readers might expect reading about it in this context. [http://bigwww.epfl.ch/publications/thevenaz9801.html]. Last but not least, the authors must be commended for the related data and code publication on Zenodo and Github - this is exactly how image data science is supposed to be published.

Comments for the author

I do have two major concerns about the way how the manuscript is written:
* Instead of describing the datasets and applied procedures so detailed (listing all parameters in the format "parameter=value"), an explanation might be helpful how a user can tune parameters when correcting drift in their own data; a quick guide how to use the tool. Details can be written in the supplementary materials or on the github website.
* I could imagine that restructuring the text a bit more clearly into "Methods", "Results" and sub-sections could help the reader. E.g. if the reader wanted to assess how registration quality was measured, it is a bit hidden at multiple places in the text.

And two more things:
* I quickly scanned through the github repository and the ImageJ Macro code. The code quality is in general ok, but some files appear to have issues with correct indentation. Sometimes closing curly brackets are hidden at the end of a line and sometimes at the beginning of a line. Example: https://github.com/guijacquemet/Fast4DReg/blob/3ea546c6c77fc197468008428d4956ad394d1524/Fast4DReg/macros/time_apply.ijm#L130 and https://github.com/guijacquemet/Fast4DReg/blob/3ea546c6c77fc197468008428d4956ad394d1524/Fast4DReg/macros/time_apply.ijm#L250-L251 There are tools for beautifying code, which I highly recommend for making code publication-ready: https://beautifier.io/
* In case I shall review a second version of the manuscript, it would be great to have some line numbers printed on the PDF.

While reading the manuscript, I took some minor notes below. All of those are just thoughts of a reviewer and may be ignored in case I missed the right context.

- Introduction

"... due to the microscopy instability caused, [...], by ..." consider rephrasing. I'm not sure if instability of the microscope is the right term here.

"... channel registration is a crucial processing step [...] including colocalization analysis" This sentence is a bold statement leaving a couple of attack angles open. For example: is it scientifically ok to register channels before analyzing colocalization between channels? Consider rewriting this sentence and focusing more on drift correction.

"ITK": Write out the "Insight ToolKit" before using its abbreviation.

"... we felt limited by [...] available features" I'm curious, would it be possible to mention such a key feature that's missing?

"Fast4DReg is fast and has a simple graphical interface." Would it be possible to show the interface in a Figure? The reader could then judge how simple the interface is. Also I would consider rephrasing, instead of "simple" (which has a negative sound) one could use "easy-to-use".

- Implementation, operation and test datasets

Consider renaming this section to "Methods" and introducing sub-sections such as "Datasets", "Algorithms", "Metrics".

"Fast4DReg breaks the drift correction task into two steps: First, estimation of drift followed by applying the drift correction." I think it would be good to introduce three terms in this context, which are common among computer folks and sometimes wrongly used among non-computer folks:

© 2023. Published by The Company of Biologists under the terms of the Creative Commons Attribution License (https://creativecommons.org/licenses/by/4.0/).

* Image registration: Determination/estimation of a transformation that corrects the drift optimally.
* Image transformation: Applying the determined parameters to produce a corrected image.
* Translation: A specific image transformation where pixel intensities are moved along a vector. All pixels move the same distance in the same direction.

"..., the drift plots, a drift table,..." this sounds like these terms were introduced before, but they were not.

"the movie's previous frame" consider rephrasing - a movie does not have a previous frame - maybe something like "subsequent frames".

"cross-correlation only works well to register channels where similar structures/cells are labeled." this brings us back to my earlier comment. If we wanted to analyze colocalization, we should not use Fast4DReg to register images; or only with a calibration slide. I see two options: write more precisely and guide users to not do wrong things with their data; and/or: focus on timelapse-registration and mention the colocalization-application less prominently.

"Fast4DReg expects as input one or multiple single channel 2D or 3D video and outputs corrected files, ... that can be applied to other channels as needed." consider splitting this sentence into two or three. I had to read it multiple times. The application to other channels is a text duplication to the previous pipeline section. I love this feature, but it doesn't need to mentioned twice.

"... are available on the Fast4DReg GitHub page." write out the link at least once, so that also people who read the printed paper version can get an idea where to find it online.

Test datasets:

"HUVEC" What is this?

"a registration slide -dataset" I'm not sure what this is. Is it a calibration slide?

Dataset 1:

"..., one with only a small amount of drift, and one with a large drift..." Is it possible t o specify the drift in microns and/or pixels? I think this could be done in a single sentence and the math in the next section could be removed. Image translation and Gaussian noise are more trivial than the explanation using the three equations and the sentences after. Instead of describing it so detailed, the authors could point to a macro which does that in the supplementary materials.

"Fast4DReg: xy-projection type = max intensity, xy time averaging = 1, x...", "Correct 3D drift: Channel for registration = 1, Multi time scale computing = enabled,...", "Fijiyama: Max subsampling factor = 4, Min subsampling factor = 2, ..." this sounds like it would be better expressed as a table; potentially in the supplementary materials.

"The drift correction performance was then quantified by measuring the Pearson’s correlation coefficient between frames" I'm curious: Why wasn't the determined transformation compared to the ground-truth transformation? This could give us an exact measure of misalignment.

Dataset 2/3/4: See my comments above. I think the exact parameters applied for the registration might not be necessary for the main manuscript text.

- Use cases

Consider renaming this section to "Results"

"To assess the capabilities of Fast4DReg to correct 3D videos, we compared Fast4DReg results to two other widely used state-of-the-art drift correction methods available in Fiji ... For this purpose, three synthetic videos with known amounts of drift were created: one with no drift, one

© 2023. Published by The Company of Biologists under the terms of the Creative Commons Attribution License (https://creativecommons.org/licenses/by/4.0/).

displaying a small amount of drift, and another with a larger amount of drift" this was mentioned above already; the authors could phrase it much shorter as a recap here.

"... a perfect drift correction will generate identical time frames." If I understood the method correctly, also noise was added to the dataset. Thus identical images can hardly be achieved.

"To carefully quantify the performance of these three software, we selected a z-slice and ..." this sounds like a method description that belongs in the section before.

"This is perhaps because part of the data goes out of the imaging volume several times." This sounds like it would be better mentioned in the discussion section.

"To obtain a more quantitative estimate of the performance of Fast4DReg [...] we measured Pearson’s correlation coefficients." Consider introducing a "Quality metrics" section above and collect all used metrics there.

Figure 3: In this figure it says "Correct3DD" and I like it. It's easier to understand than when "Correct 3D drift" is mentioned in a sentence. Consider introducing abbreviations like this at the very beginning and then using it in the entire manuscript.

Page 7:

"Our primary motivation behind developing Fast4DReg was to create a 3D registration pipeline that is easy to use, ..." it sounds like there starts a new section here. I'm not sure what headline this section could have. Consider moving the thoughts mentioned here in the discussion section.

"... to process the HUVEC dataset using two different computers. We found that Fast4DReg (1 min 48s to 6 min 24s) is 4-7 times faster than Correct 3D drift (12 min 30 to 24 min) and 20 to 70 times faster than Fijiyama (1 h to 2h) when correcting the HUVEC dataset (Figure 3d)." I'm not sure how the two different computers are relevant in this context. Consider writing all these numbers in a table to make the differences more clear.

"These differences are significant ..." When the term "significant" comes up, I always expect a statistical test. Was one applied here?

"Fast4DReg speed is likely due to two factors: ..." this sounds like content for the discussion section.

"In this dataset, the raw images display significant xy and z misalignment due to chromatic aberrations..." this sounds like it belongs in the Methods/Datasets section some pages above.

"we envision that the approach described here can be used" this might also better fit in the Discussion or even Conclusion section.

"Despite its performance, Fast4DReg has several limitations." I love this section. Big thanks to the authors for this.

"But using 3D cross-correlation will likely impede processing times." I agree. GPU-acceleration might be necessary :-)

"To promote adoption by the community, Fast4DReg is available through GitHub/Zenodo, where the pipeline, test datasets, and detailed step-by-step instructions are provided." This code/data publication is an excellent example of how bio-image analysis method development publications are supposed to be. Fantastic work.

© 2023. Published by The Company of Biologists under the terms of the Creative Commons Attribution License (https://creativecommons.org/licenses/by/4.0/).

First revision

Author response to reviewers' comments

Reviewer 1

Reviewer 1 Advance Summary and Potential Significance to Field:
The authors present Fast4DReg, a collection of scripts for the popular FIJI platform for the correction of axial and lateral drift in time-lapse imaging datasets. Fast4DReg can also be used to correct misalignment between channels in multi-channel datasets. The principles underlying the software's operation are illustrated with some nice schematics and the authors use a variety of test datasets to test the performance of their software against other state-of-the-art tools. While the performance of Fast4DReg appears to be comparable to another freely-available tool (Correct 3DD), the results presented suggest that Fast4DReg is significantly faster, which would undoubtedly make Fast4DReg of interest to the broader community. However, there are some shortcomings associated with the demonstration of the software that should be addressed to strengthen the case for the software's adoption.

We thank the reviewers for the positive and constructive feedback on our manuscript. The comments helped us to considerably improve our manuscript.

Reviewer 1 Comments for the Author:
Firstly, while captured as 3D image stacks, the test datasets used are largely two-dimensional in nature. For example, the registration slide dataset is a two-dimensional array of dots. It would be useful to know how Fast4DReg performs on truly 3D data and how it compares to other software on such datasets.

We are unsure why the reviewer indicates that our test datasets are two-dimensional. All of our test datasets are three-dimensional and contain 3D structures. For instance, our synthetic dataset is an AsPC1 cell (pancreas adenocarcinoma cell line) expressing Lifeact-mScarlet-I migrating inside the vasculature of a zebrafish embryo. The HUVEC monolayer dataset was imaged at a high enough resolution to make the ventral and dorsal actin structures visible. The SIM datasets used to demonstrate the channel alignment capabilities are also 3D. Nevertheless, in the revised version of our manuscript, we provide two additional test datasets.

1) One live imaging dataset where cancer cells are migrating inside the lung vasculature (see new Fig. 5 and Movies 4 and 5).
2) A new 3D SIM dataset (see new Fig. 6).

Fast4DReg also performed well when registering these two datasets.

© 2023. Published by The Company of Biologists under the terms of the Creative Commons Attribution License (https://creativecommons.org/licenses/by/4.0/).

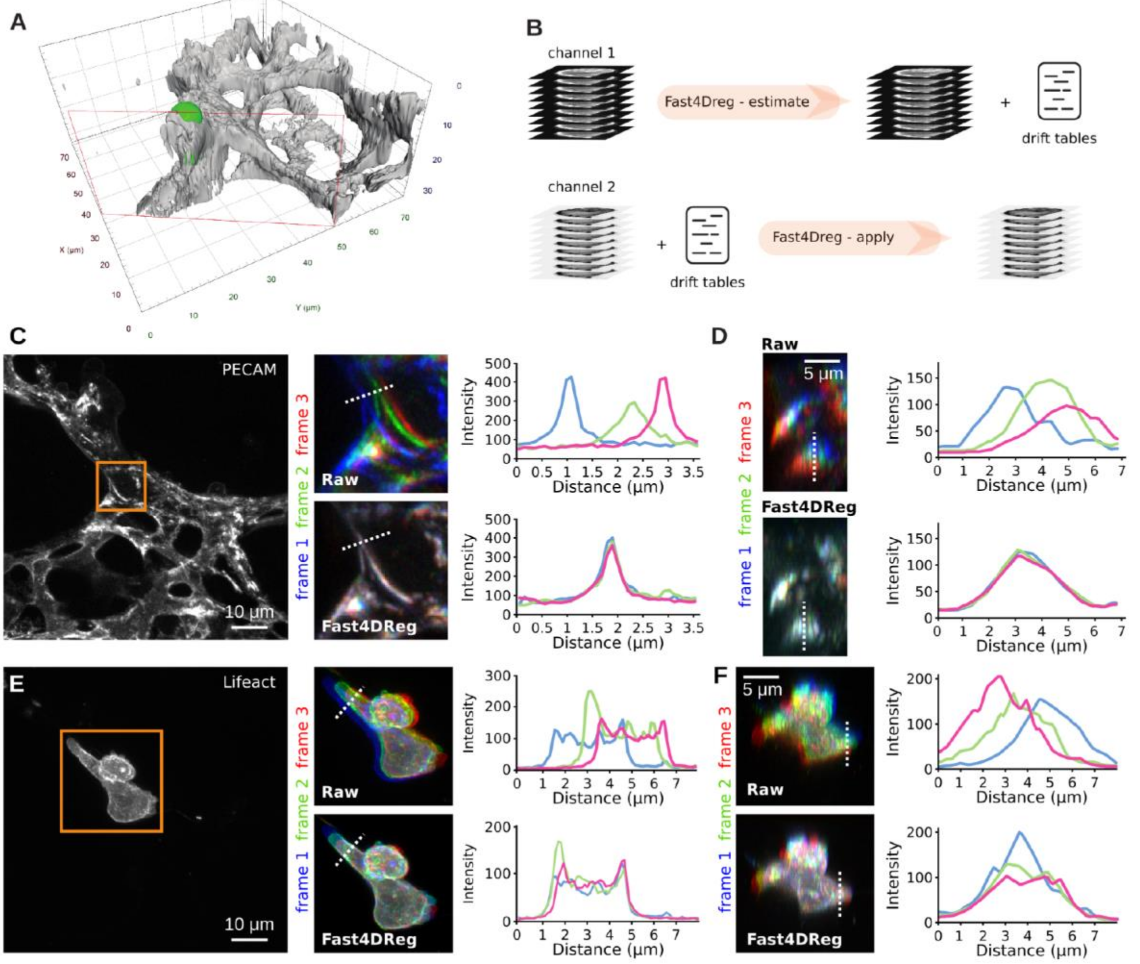

Figure 5: Registration of a 3D multicolor video using Fast4DReg.
A 3D multicolor video of cancer cells migrating inside the lung vasculature was corrected using Fast4DReg. A) 3D surface rendering of a selected time point (see also video 4). The rendering was created using Arivis Vision4D. B) Cartoon illustrating the pipeline used to correct a multicolor 3D video using Fast4DReg. For this dataset, the drift was first estimated using the vasculature images (channel 1), and the resulting drift table was then applied to the cancer cell images (channel 2). C-E) Three consecutive frames of the vasculature (C) and cancer cell images (D) were pseudo-colored blue, green, and red and merged. White indicates structural overlaps between the three frames. Line profiles to further study the overlap between frames were drawn as shown. C and E) Z-projections are displayed to visualize the lateral misalignments corrected by Fast4DReg. Scale bars = 10 µm. D and F) Y-projections are displayed to visualize the axial misalignment corrected by Fast4DReg. Scale bars = 5 µm.

© 2023. Published by The Company of Biologists under the terms of the Creative Commons Attribution License (https://creativecommons.org/licenses/by/4.0/).

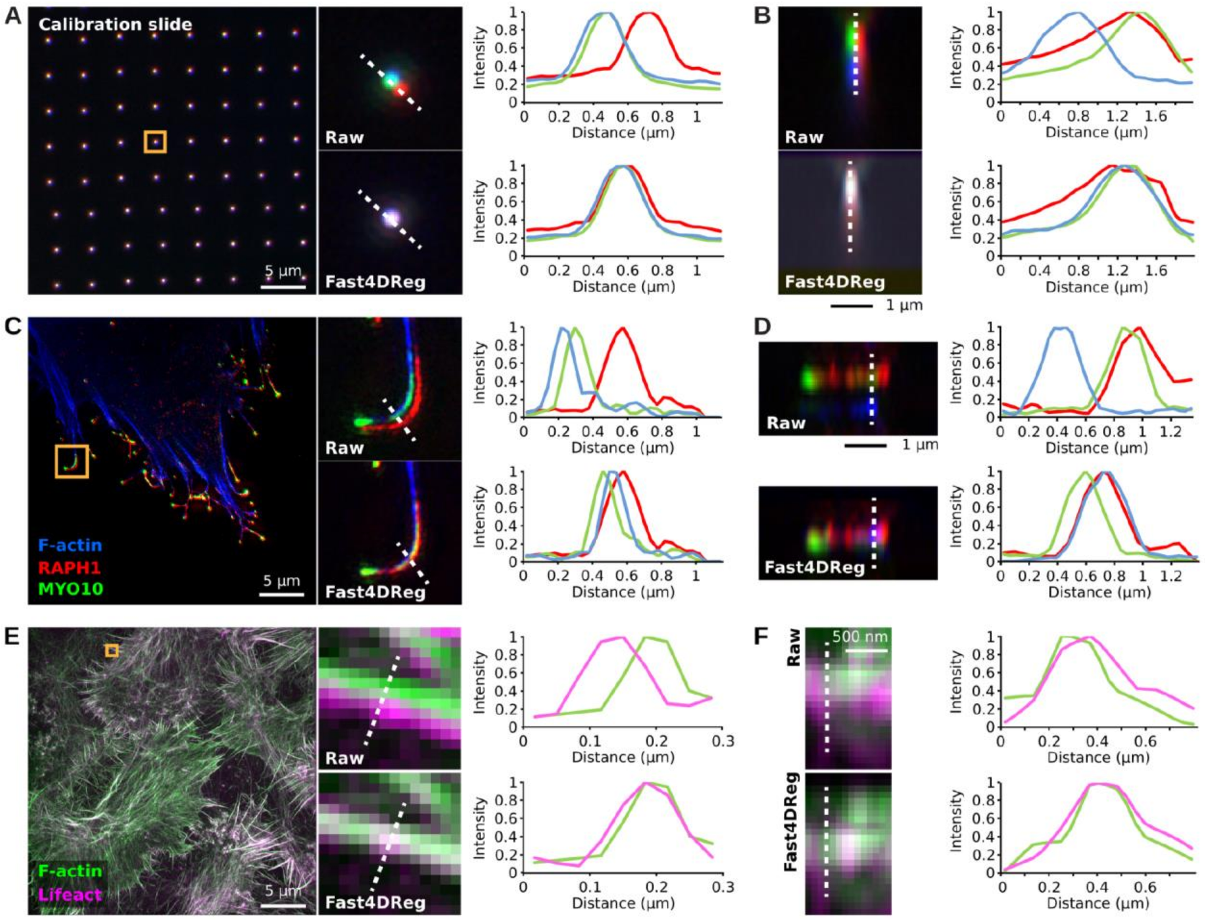

Figure 6: Fast4DReg can align 3D multi-channel images.
A-B) A 3-channel 3D calibration slide image was aligned using Fast4DReg. Merged images and line intensity profiles are displayed to highlight the level of overlap between the three channels. Scale bar = 5 µm. A) A z-projection is displayed to visualize the lateral misalignment corrected by Fast4DReg. B) A y-projection of one of the calibration slide spots is displayed to illustrate the axial misalignment corrected by Fast4DReg. C-D) The drift table generated in (A-B) was then used to correct a 3D SIM image of a U2-OS cell expressing GFP-tagged Lamellipodin (RAPH1, red), MYO10-mScarlet (green), and labeled to visualize its actin cytoskeleton (blue). C) A z-projection is displayed to visualize the lateral misalignment, evident in filopodia, corrected by Fast4DReg. Scale bar = 5 µm. D) A y-projection of one filopodium visualizes the axial misalignment corrected by Fast4DReg. Scale bar = 1 µm. E-F) A 3D SIM image of MCF10DCIS.com cells expressing RFP-Lifeact (magenta) and stained to visualize F-actin (green) was aligned using Fast4DReg directly. E) A z-projection is displayed to visualize the lateral misalignment corrected by Fast4DReg. Scale bar = 5 µm. F) A y-projection visualizes the slight axial misalignment corrected by Fast4DReg. Scale bar = 500 nm.

Secondly, there is a lack of detail concerning the algorithmic underpinnings of Fast4DReg. More details on how the cross-correlation matrices are calculated is warranted, given how central this is to the method. For example, what correlation coefficient is used? And how is the peak determined? How does Fast4DReg discriminate between multiple peaks in the CCM?

We thank the reviewer for bringing this forward. We now provide additional information concerning the algorithmic underpinnings Fast4DReg in the method section. Also provided below:

“To estimate the lateral or axial drift of a 3D video, Fast4DReg creates z or y intensity projections for each time point to create a 2D video. Fast4DReg then estimates the linear drift between the reference and moving frames by calculating their cross-correlation matrix (CCM). In Fast4DReg, in a similar fashion to the work of Sun, H. (Sun, 2002), the cross-correlation between two images is calculated by performing the Discrete Hartley Transform (DHT) on both images, followed by a

© 2023. Published by The Company of Biologists under the terms of the Creative Commons Attribution License (https://creativecommons.org/licenses/by/4.0/).

multiplication of one of the transformed images by the complex conjugate of the other. The result of this multiplication is then inversely transformed back to real space, generating the cross-correlation map (CCM). A bicubic spline interpolation is then used to upscale the CCM and achieve subpixel precision. The upscaled CCM is normalized by calculating the Pearson's Correlation Coefficient between the two images shifted according to the minimum and maximum values of the upscaled CCM. Finally, the linear shift between the two images is then calculated by taking the global maximum peak of the normalized up-scaled CCM [as demonstrated by (Laine et al., 2019)]."

Finally, in quantifying the registration accuracy of Fast4DReg relative to other tools, the authors rely solely on Pearson's Correlation Coefficient. Is this the same correlation coefficient used to construct the CCMs? If so, it is perhaps not surprising that Fast4DReg performs so well! How do the various tools compare when registration is evaluated using other metrics?

We thank the reviewer for bringing this critical point forward. As described above, Pearson's Correlation Coefficient is used by Fast4DReg to normalize the CCM. This would, however, not influence the validity of using Pearson's Correlation Coefficient to compare the similarity of images post-registration. However, the reviewer's point is well taken, and relying on a single metric to assess image similarities is dangerous. We now provide three additional image similarity metrics, namely peak signal-to-noise ratio (PSNR), structural similarity index measure (mSSIM), and normalized root mean squared error (NRMSE), for our test datasets (all described in the method section). Interestingly the PSNR metric is the most sensitive to minor misalignment and highlights more significant differences between Fast4DReg and the other tool tested. Importantly, Fast4DReg outperforms the other tool tested regardless of the metric used.

Other General Points

* I have reservations about "advertising" this tool as a means to correct misalignment between channels. This could lead to the artificial localisation of signals that are not in fact co-localised at all. The authors should stress that this functionality should only be used for co-labelled structures (such as fluorescent beads or calibration slides). A note of caution is sounded, but I think this needs to be expanded upon. Using a calibration slide should always be the recommended approach for correcting channel misalignment.

We thank the reviewer for bringing this forward. We now stress the importance of using calibration slides to register misaligned channels, especially when performing colocalization analyses. We also provide another example where direct alignment can be useful: alignment of images with the same structures labeled with different dyes (see new Fig. 6).

* How do the authors suggest users differentiate between drift and sample migration?

The reviewer is highlighting a crucial point. Indeed movement of biological samples is not always undesirable, and recording such movements may be the objective of the microscopy experiment. For instance, we routinely record cell migration using live-cell imaging with the aim of tracking and quantifying cell movement. In this case, using a drift correction algorithm may be counter-productive. However, the overall impact of the drift correction algorithm depends on the acquisition speed, the sample movement speed, and the amount of drift to be corrected. Correcting the drift using the previous frame as a reference should eliminate sample drift without impacting the overall sample movement if the acquisition speed is faster than the sample movement. When the acquisition speed is not fast enough, the experimenter will have to decide if correcting the drift is worth it. This will likely depend on the amount of drift to be corrected.

Of course, if highly mobile structures need to be imaged and drift corrected, another channel could be used to estimate the drift. The estimated drift can then be used to correct the channel containing the highly motile structures.

* How would it handle very large datasets? It is stated that "Fast4DReg has a RAM-saving mode that allows the registration of large datasets" (and "large 3D videos" are referenced in the abstract), but none of the test datasets are particularly large. It is also stated that this RAM-saving mode makes processing times "slightly longer", but this is not quantified - this is an important omission, given how processing speed is one of the main advantages of Fast4DReg.

© 2023. Published by The Company of Biologists under the terms of the Creative Commons Attribution License (https://creativecommons.org/licenses/by/4.0/).

We thank the reviewer for bringing this point forward. Indeed, the available RAM will still limit the dataset size that can be processed using Fast4DReg. We now toned down the text and removed the mention of "large datasets" to ensure the reader is not misinformed. In the new release, we also optimized Fast4DReg RAM saving mode, which now runs slightly faster than the normal mode. We provide quantifications for the RAM-saving mode processing times in the revised manuscript (See new Fig. S1D).

D

	Computer 1	Computer 2
**Fast4DReg**	1.4 min	5.9 min
**Fast4DReg (RAM saving mode)**	1.15 min	4.5 min

Figure S1: Fast4DReg outperforms Correct3DD and Fijiyama on a synthetic 3D + t dataset

D) Comparison of Fast4DReg processing time when the RAM saving mode is enabled (HUVEC monolayer -dataset).

* Correlation Coefficients (particularly Pearson's) are sensitive to noise - how well does Fast4DReg function on datasets with a low signal-to-noise ratio?

This is an interesting point; we have now addressed it in detail. We created 12 synthetic datasets with different signal-to-noise ratios. We then tested the ability of Fast4DReg to correct them (see new Fig. 3). These analyses indicate that Fast4DReg is not affected by noise when the signal-to-noise ratio (SNR) is above 2. When the SNR is below 2, a decrease in performance can be observed. Interestingly, when the images start to be very noisy (SNR below 2), Fast4DReg performs much better when using average intensity projections instead of maximal intensity projections (Fig. 3C-E).

© 2023. Published by The Company of Biologists under the terms of the Creative Commons Attribution License (https://creativecommons.org/licenses/by/4.0/).

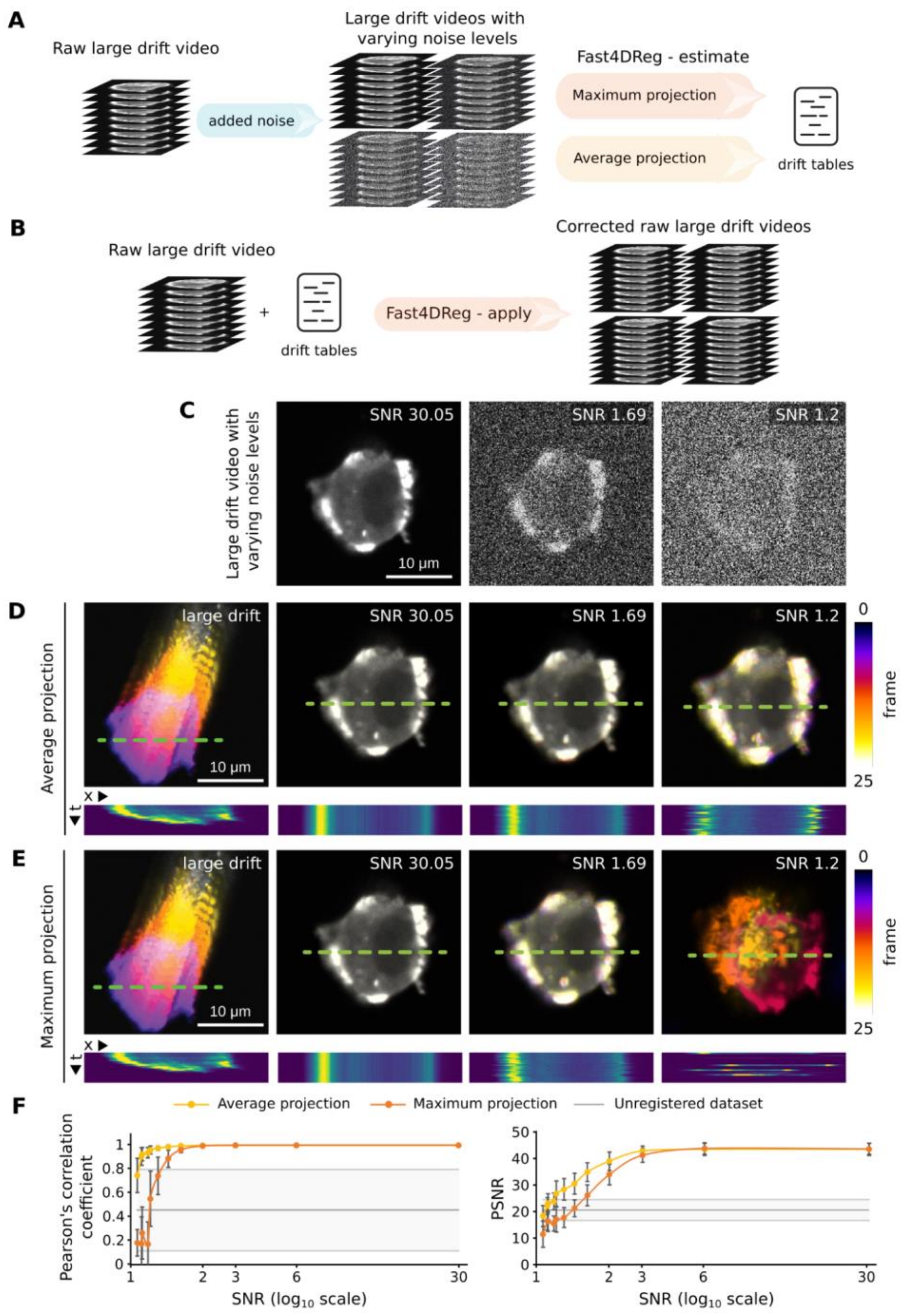

Figure 3: Fast4DReg is relatively resistant to noise.
Twelve synthetic 3D video datasets with varying amounts of noise were created and corrected using Fast4DReg, either using maximal or average intensity projections. The drift tables were then applied to the original data to assess drift correction accuracy. A-B) Cartoon illustrating the pipeline used to assess Fast4DReg sensitivity to noise. C) Example of three noisy datasets used to assess Fast4DReg sensitivity to noise. D-E) Fast4DReg drift correction performance for three noisy datasets (C) was assessed using temporal color projections of a selected z-slice (middle of the cell) and kymographs (along the green dashed line). F) Fast4DReg drift correction performance for the twelve noisy datasets was assessed using image similarity metrics. The PSNR and Pearson's correlation coefficients between the first and subsequent frames were calculated for each noise amount. For all panels, the scale bar = 10 μm. Abbreviation: Signal to noise ratio (SNR).

D) For each noise amount, the PSNR and Pearson's correlation coefficients between the first and each subsequent frame were calculated. For all panels, the scale bar = 10 μm.

© 2023. Published by The Company of Biologists under the terms of the Creative Commons Attribution License (https://creativecommons.org/licenses/by/4.0/).

Specific Points:

Introduction
* With regard to the opening paragraph, how prevalent is this problem of drift? If the microscope and sample are properly equilibrated, drift due to temperature change should be minimal, especially if the microscope in question is equipped with hardware-based drift-correction tools (such as Nikon's PFS).

Despite using similar systems as described by the reviewer, most of our live imaging datasets need to be corrected.

* "Multiview Reconstruction, which was designed to register large lightsheet fluorescence microscopy datasets, uses beads and/or segmented structures in the imaging volume to perform the registration" - this is not true. Registration can be accomplished in the absence of landmarks. Also worth noting that this registration is performed in 3D, albeit (typically) with downsampled images.

We apologize if our statement was incorrect. We, however, can identify neither in the documentation nor in the software itself if/how Multiview Reconstruction can perform landmark-free registration. To the best of our knowledge, Multiview Reconstruction requires identifying interest points before performing the registration (as stated in the documentation and tutorials). We have now rephrased our statement to reflect the documentation more accurately:

“Multiview Reconstruction, which was designed to register large light-sheet fluorescence microscopy datasets, uses interest points (e.g., fluorescent beads, nuclei, or membrane markers) in the imaging volume to perform the 3D registration, which are not always available (Preibisch et al., 2010, 2014)”

Implementation, operation, and test datasets
* What is the origin of the original image in Dataset 1?

We now provide this information in the methods.
“The original image is an AsPC1 cell (pancreas adenocarcinoma cell line) expressing Lifeact-mScarlet-I migrating inside the vasculature of a zebrafish embryo.”

* It seems a bit odd to include the computer specifications in their current location (under Dataset 2) - do these specifications only apply to Dataset 2?

Yes, we indicated the computer specifications here as we used these specific computers to measure the algorithms’ speed. We used several other computers (including these two) to register the other test datasets.

* "The Filopodia-dataset (1024x1024 px, 17 z-slices, 3 channels) consists of a 3D structured illumination microscopy (SIM) image of a U2OS cell expressing a GFP-tagged Lamellipodin fragment, MYO10-mScarlet, and labeled to visualize its actin cytoskeleton using sirActin" - this is worded confusingly. I'm not completely sure what cell structures are labelled with what fluorophores.

We have now rephrased this sentence (of note, this particular dataset has now been replaced).

“The Filopodia-dataset (1024x1024 px, 17 z-slices, 3 channels) consists of a 3D SIM image of a U2-OS cell expressing GFP-tagged Lamellipodin, MYO10-mScarlet, and labeled with SiR-actin (Spirochrome) (Miihkinen et al., 2022).”

* Dataset 2: the drift in this dataset looks very odd and periodic in nature? Could the authors explain the source of this effect?

Unfortunately not, this is a real experimental dataset, and we do not know why the drift is behaving like this here. Our best guess is that the laser-based stabilization system misbehaved during the acquisition.

© 2023. Published by The Company of Biologists under the terms of the Creative Commons Attribution License (https://creativecommons.org/licenses/by/4.0/).

* Dataset 4: similar to the above, the channel misalignment here seems excessive?

Here the channel misalignment is due to chromatic aberrations and the fact that each channel is acquired using different cameras which are not perfectly aligned. Post-acquisition realignment is, therefore, essential.

Use Cases

* "To carefully quantify the performance of these three software, we selected a z-slice..." - how? And why?

We selected the z-slice in the middle of the cell. Selecting a z-slice allowed us to evaluate the registration performance of the tools and, importantly, to understand the image similarity metrics results.

* In Fig 2C, is this a projection over time? Based on the figure legend, this would appear to be the case, but the description is confusing. For example, "This projection takes the standard deviation of the pixel intensities through the stack" suggests that we are looking at a Z-projection.

It is indeed a projection over time. We have now rephrased the figure legend to clarify.

* In Fig 2D, the standard deviation is calculated on a slice-by-slice basis - why? Why not calculate the SD over the entire volume through time at once?

We calculate the SD on a slice-by-slice basis as it allows us to better appreciate the metric's variability through the volume.

* The differences shown in Fig 2F are tiny - I don't think Pearson's is the best metric to use here, as the images contain large areas of background (which is undoubtedly skewing the correlation coefficients upwards). The contrast with Fig 3c is stark, although the two datasets are obviously quite different in nature (one is time-lapse, the other is not)

We now provide three other metrics to quantitatively assess the registration of our test datasets (as described above, Fig. S1 and Fig. 2). In particular, The PSNR metric appears well-suited to identify small registration errors (see new Fig. 2D).

* I'm not sure I understand the point of Fig 3A? It seems highly selective (spatially and temporally) and is made somewhat redundant by subsequent panels

This panel is indeed very selective, but it allows us to carefully evaluate the drift correction capabilities of Fast4DReg by checking the temporal overlap of a static feature. We believe that it is essential to combine careful visual assessment with quality metrics to evaluate the strength and weaknesses of a tool.

* Similarly, Fig 3B is confusing - it's pretty difficult to tell if Fast4DReg is better or worse (or comparable?) than Correct 3DD based on these kymographs. Also, how was the location in Z selected for the kymograph? Is it the mid-point of the stack?

We thank the reviewer for highlighting this point. The Z-plane selected corresponds to the monolayer ventral plane (which is close to the stack center). As described in the text, these kymographs highlight that the movie corrected by Correct 3DD is slowly sinking over time.

* "In the original data, the kymograph shows a clear band pattern due to the microscope stage jumping cyclically." - why is the microscope stage doing this?!?

As noted above, we do not know for sure. We suspect that the laser-based stabilization system misbehaved during the acquisition.

* "Indeed, we consider that efficient drift correction will make successive frames more similar to one another despite the inevitable biological changes." - this is obviously a problem that the authors should elaborate on

© 2023. Published by The Company of Biologists under the terms of the Creative Commons Attribution License (https://creativecommons.org/licenses/by/4.0/).

We added the following sentence to clarify the issue:

“Indeed, efficient drift correction will make successive frames more similar despite the inevitable biological changes. These biological changes will, however, lead to lower image similarity metrics than the one measured with our synthetic dataset (Fig. 2 and 3), even if the data is perfectly registered.”

* "Using this metric, we find that Fast4DReg performed equally or slightly better than Correct 3DD drift on this dataset" - this statement should be more quantitative. What does "equally" or "slightly better" mean?

We have now rephrased this statement:

“Using these metrics, we find that Fast4DReg and Correct3DD improve adjacent frame similarity on this dataset and perform similarly (Fig. 4C)”

* "We chose images of cells displaying filopodia as these actin structures are very thin and narrow, rendering them almost two-dimensional" - what happens if such structures are not available? The authors touch on this problem by stating that "This approach will not work with all images, as the registration method used here will work only if sufficient structural overlap exists between the channels", but then go on to state that they "envision that the approach described here can be beneficial in correcting channel misalignment when no calibration data is available". As stated above, using a calibration slide should always be the recommended approach for correcting channel misalignment to avoid apparent co-localisation of signals that are not, in reality, co-localised.

We fully agree with the reviewer, and we have now added the following sentences to the manuscript:

“Combined with the Fast4DReg batch processing mode, we envision that the indirect channel alignment approach described here will be advantageous when performing colocalization analyses.”

And “Additionally, we do not recommend directly aligning images aimed for colocalization analysis as it may lead to artefactual results.”

Reviewer 2

Reviewer 2 Advance Summary and Potential Significance to Field:
I had the pleasure to review a manuscript titled "Fast4DReg: Fast registration of 4D microscopy datasets" by Joanna W. Pylvänäinen et al. The manuscript describes a 3D/4D image registration tool tailored to correct drift in microscopy timelapse imaging data. The presented problem is highly relevant and the demonstrated solution is a fantastic achievement - in particular in the context of processing speed and user-friendlyness. The manuscript is written in excellent english and has a clear red line. The Figures are outstandingly well prepared and the authors successfully explain a highly complex topic in an accessible fashion. I am fully convinced that the authors give a new powerful tool into the hands of end-users. Practically speaking, Fast4DReg makes a functionality available to ImageJ/Fiji users that was not available yet and highly demanded. The most commonly used tool for similar purposes (StackReg) is available for 2D images only and lacks substantial features the authors implemented in Fast4DReg. In this context, I would like to highlight a sentence from the pipeline section: "Notably, the drift table can then be applied to correct other images using the same parameters" This feature is super amazing and presumably response to user requests. I have heard StackReg users a thousand times complaining that exactly this feature is not available. Btw. StackReg could be cited as well as readers might expect reading about it in this context. http://bigwww.epfl.ch/publications/thevenaz9801.html]. Last but not least, the authors must be commended for the related data and code publication on Zenodo and Github - this is exactly how image data science is supposed to be published.

We thank the reviewer for the very positive feedback on our work and for supporting publication. The comments helped us greatly improve our manuscript.

© 2023. Published by The Company of Biologists under the terms of the Creative Commons Attribution License (https://creativecommons.org/licenses/by/4.0/).

Reviewer 2 Comments for the Author:

I do have two major concerns about the way how the manuscript is written:
* Instead of describing the datasets and applied procedures so detailed (listing all parameters in the format "parameter=value"), an explanation might be helpful how a user can tune parameters when correcting drift in their own data; a quick guide how to use the tool. Details can be written in the supplementary materials or on the github website.

We thank the reviewer for this important suggestion.

1) We have updated and improved our guide on how to use Fast4DReg, located on our GitHub page (https://github.com/guijacquemet/Fast4DReg).
2) All the software parameters used to correct the datasets presented in this manuscript are now available in Table S1.

* I could imagine that restructuring the text a bit more clearly into "Methods", "Results" and sub-sections could help the reader. E.g. if the reader wanted to assess how registration quality was measured, it is a bit hidden at multiple places in the text.

We thank the reviewer for suggesting this. We have now fully restructured the text.

And two more things:
* I quickly scanned through the github repository and the ImageJ Macro code. The code quality is in general ok, but some files appear to have issues with correct indentation. Sometimes closing curly brackets are hidden at the end of a line and sometimes at the beginning of a line.
Example:
https://github.com/guijacquemet/Fast4DReg/blob/3ea546c6c77fc197468008428d4956ad3
94d1524/Fast4DReg/macros/time_apply.ijm#L130 and
https://github.com/guijacquemet/Fast4DReg/blob/3ea546c6c77fc197468008428d4956ad3
94d1524/Fast4DReg/macros/time_apply.ijm#L250-L251

There are tools for beautifying code, which I highly recommend for making code publication-ready:
https://beautifier.io/

We thank the reviewer for checking the code quality. We have now improved its readability.

In case I shall review a second version of the manuscript, it would be great to have some line numbers printed on the PDF.

We now provide line numbering in the revised manuscript.

While reading the manuscript, I took some minor notes below. All of those are just thoughts of a reviewer and may be ignored in case I missed the right context.

- Introduction

"... due to the microscopy instability caused, [...], by ..." consider rephrasing. I'm not sure if instability of the microscope is the right term here.

We have now rephrased this sentence to:
"Drift can be caused, for example, by temperature changes leading to thermal expansion of the microscope mechanical components or by the movement of the sample itself."

"... channel registration is a crucial processing step [...] including colocalization analysis" This sentence is a bold statement leaving a couple of attack angles open. For example: is it scientifically ok to register channels, before analyzing colocalization between channels? Consider rewriting this sentence and focusing more on drift correction.

We thank the reviewer for highlighting this critical point. Yes, it is useful to register channels before performing colocalization analysis. However, as rightly highlighted by the reviewers, this

© 2023. Published by The Company of Biologists under the terms of the Creative Commons Attribution License (https://creativecommons.org/licenses/by/4.0/).

should be done using calibration slides. We now clarify this in the introduction.

"ITK": Write out the "Insight ToolKit" before using its abbreviation.
Thanks, done.

"... we felt limited by [...] available features" I'm curious, would it be possible to mention such a key feature that's missing?

Correct3DD is a very powerful Fiji plugin that we use routinely, but as stated in the text, we felt limited by key features:

- Correct3DD Is relatively slow
- Correct3DD can be quite inaccurate on some of our datasets.
- Correct3DD cannot perform axial drift correction alone.
- Correct3DD Drift tables are not saved and cannot be applied to other datasets.
- Correct3DD is not capable of correcting misaligned channels (directly or indirectly).
- Batch mode is only available using scripting.

"Fast4DReg is fast and has a simple graphical interface." Would it be possible to show the interface in a Figure? The reader could then judge how simple the interface is. Also I would consider rephrasing, instead of "simple" (which has a negative sound) one could use "easy-to-use".

We thank the reviewer for these suggestions. We have now included them in our revised manuscript (Fig. S2).

- Implementation, operation and test datasets

Consider renaming this section to "Methods" and introducing sub-sections such as "Datasets", "Algorithms", "Metrics".

We thank the reviewer for this suggestion. It is now incorporated into our revised manuscript.

"Fast4DReg breaks the drift correction task into two steps: First, estimation of drift followed by applying the drift correction." I think it would be good to introduce three terms in this context, which are common among computer folks and sometimes wrongly used among non-computer folks:
* Image registration: Determination/estimation of a transformation that corrects the drift optimally.
* Image transformation: Applying the determined parameters to produce a corrected image.
* Translation: A specific image transformation where pixel intensities are moved along a vector. All pixels move the same distance in the same direction.

Done.

"..., the drift plots, a drift table,..." this sounds like these terms were introduced before, but they were not.

We now explain these terms in the main text.

"the movie's previous frame" consider rephrasing - a movie does not have a previous frame - maybe something like "subsequent frames".

We now rephrase it to “consecutive frames.”

"cross-correlation only works well to register channels where similar structures/cells are labeled." this brings us back to my earlier comment. If we wanted to analyze colocalization, we should not use Fast4DReg to register images; or only with a calibration slide. I see two options: write more precisely and guide users to not do wrong things with their data; and/or: focus on timelapse-registration and mention the colocalization-application less prominently.

As described above, we chose the first option and described more precisely how to use Fast4DReg before colocalization analysis. We also provide a new example highlighting this in the main figures

© 2023. Published by The Company of Biologists under the terms of the Creative Commons Attribution License (https://creativecommons.org/licenses/by/4.0/).

(See new Fig. 6).

"Fast4DReg expects as input one or multiple single channel 2D or 3D video and outputs corrected files, ... that can be applied to other channels as needed." consider splitting this sentence into two or three. I had to read it multiple times. The application to other channels is a text duplication to the previous pipeline section. I love this feature, but it doesn't need to mentioned twice.

Thank you. We have now improved this section.

"... are available on the Fast4DReg GitHub page." write out the link at least once, so that also people who read the printed paper version can get an idea where to find it online.

The link is now available in the “Software Availability” part of the method section.

Test datasets:

"HUVEC" What is this?

Thank you for noticing this. We now explain this abbreviation in the text.

“We used a long 3D video of a human umbilical vein endothelial cells (HUVEC) monolayer labeled with silicon rhodamine (SiR)-actin and imaged using an Airyscan confocal microscope”

"a registration slide -dataset" I'm not sure what this is. Is it a calibration slide?

We have now corrected our terminology throughout the manuscript.

Dataset 1:

"..., one with only a small amount of drift, and one with a large drift..." Is it possible to specify the drift in microns and/or pixels? I think this could be done in a single sentence and the math in the next section could be removed.
Image translation and Gaussian noise are more trivial than the explanation using the three equations and the sentences after. Instead of describing it so detailed, the authors could point to a macro which does that in the supplementary materials.

We thank the reviewer for this suggestion and have simplified our material and methods section. In addition, we also created a new supplementary figure that contains the actual drift table used to generate these synthetic datasets (see new Fig. S1A).

"Fast4DReg: xy-projection type = max intensity, xy time averaging = 1, x...", "Correct 3D drift: Channel for registration = 1, Multi time scale computing = enabled,...", "Fijiyama: Max subsampling factor = 4, Min subsampling factor = 2, ..." this sounds like it would be better expressed as a table, potentially in the supplementary materials.

We thank the reviewer for this suggestion. All parameters used have now been moved to Table S1.

"The drift correction performance was then quantified by measuring the Pearson’s correlation coefficient between frames" I'm curious: Why wasn't the determined transformation compared to the ground-truth transformation? This could give us an exact measure of misalignment.

We decided to use image similarity metrics as we could combine their interpretation more easily with a visual inspection of the registration results. We believe that using metrics alone or visual inspection alone is not always sufficient to properly appreciate the final results.

Dataset 2/3/4: See my comments above. I think the exact parameters applied for the registration might not be necessary for the main manuscript text.

As indicated above, all parameters used are now included in Table S1.

© 2023. Published by The Company of Biologists under the terms of the Creative Commons Attribution License (https://creativecommons.org/licenses/by/4.0/).

- Use cases

Consider renaming this section to "Results"

Done!

"To assess the capabilities of Fast4DReg to correct 3D videos, we compared Fast4DReg results to two other widely used state-of-the-art drift correction methods available in Fiji ... For this purpose, three synthetic videos with known amounts of drift were created: one with no drift, one displaying a small amount of drift, and another with a larger amount of drift" this was mentioned above already; the authors could phrase it much shorter as a recap here.

We kept this text in the new version of the manuscript as the datasets are now detailed in the method section.

"... a perfect drift correction will generate identical time frames." If I understood the method correctly, also noise was added to the dataset. Thus, identical images can hardly be achieved.

Excellent point. We now rephrased to:

“As these videos were generated by duplicating an acquired single 3D stack and adding artificial drift, a perfect drift correction will generate near identical time frames as only the background noise will differ”

"To carefully quantify the performance of these three software, we selected a z- slice and ..." this sounds like a method description that belongs in the section before.

We kept this information in the main text as we think it is important to understand the results presented in the figures.

"This is perhaps because part of the data goes out of the imaging volume several times." This sounds like it would be better mentioned in the discussion section.

We kept this point in the result section as we felt it would be too disconnected from the rest of the text if we moved it to the discussion.

"To obtain a more quantitative estimate of the performance of Fast4DReg [...] we measured Pearson’s correlation coefficients." Consider introducing a "Quality metrics" section above and collect all used metrics there.

A quality metric section is now available in the methods section.

Figure 3: In this figure it says "Correct3DD" and I like it. It's easier to understand than when "Correct 3D drift" is mentioned in a sentence. Consider introducing abbreviations like this at the very beginning and then using it in the entire manuscript.
Thank you for this excellent suggestion. We have now adopted it in the manuscript.

Page 7:

"Our primary motivation behind developing Fast4DReg was to create a 3D registration pipeline that is easy to use, ..." it sounds like there starts a new section here. I'm not sure what headline this section could have. Consider moving the thoughts mentioned here in the discussion section.

We have now simplified this section.

"... to process the HUVEC dataset using two different computers. We found that Fast4DReg (1 min 48s to 6 min 24s) is 4-7 times faster than Correct 3D drift (12 min 30 to 24 min) and 20 to 70 times faster than Fijiyama (1 h to 2h) when correcting the HUVEC dataset (Figure 3d)." I'm not sure how the two different computers are relevant in this context. Consider writing all these numbers in a table to make the differences more clear.

© 2023. Published by The Company of Biologists under the terms of the Creative Commons Attribution License (https://creativecommons.org/licenses/by/4.0/).

We thank the reviewer for this suggestion; we now present these results in a table format in Fig. 4D.

"These differences are significant ..." When the term "significant" comes up, I always expect a statistical test. Was one applied here?

We have now rephrased this sentence.

"Fast4DReg speed is likely due to two factors: ..." this sounds like content for the discussion section.

We move this part to the discussion section.

"In this dataset, the raw images display significant xy and z misalignment due to chromatic aberrations..." this sounds like it belongs in the Methods/Datasets section some pages above.

We kept this information in the main text, as the method section is now located after the results section.

"we envision that the approach described here can be used" this might also better fit in the Discussion or even Conclusion section.

We kept this sentence here as we thought that this point was too small to make an individual discussion item.

"Despite its performance, Fast4DReg has several limitations." I love this section. Big thanks to the authors for this.

We agree with the reviewer that highlighting the weaknesses is as important as highlighting the strengths. It helps users identify the most suitable tool for the job.

"But using 3D cross-correlation will likely impede processing times." I agree. GPU-acceleration might be necessary :-)

Looking forward to trying this approach in the future!

"To promote adoption by the community, Fast4DReg is available through GitHub/Zenodo, where the pipeline, test datasets, and detailed step-by-step instructions are provided." This code/data publication is an excellent example of how bio-image analysis method development publications are supposed to be. Fantastic work.

We thank the reviewer for these supporting comments!

Second decision letter

MS ID#: JOCES/2022/260728

MS TITLE: Fast4DReg: Fast registration of 4D microscopy datasets

AUTHORS: Joanna Pylvänäinen, Romain F Laine, Bruno MS Saraiva, Sujan Ghimire, Gautier Follain, Ricardo Henriques, and Guillaume Jacquemet
ARTICLE TYPE: Tools and Resources

We have now reached a decision on the above manuscript.

To see the reviewers' reports and a copy of this decision letter, please go to: https://submit-jcs.biologists.organd click on the 'Manuscripts with Decisions' queue in the Author Area. (Corresponding author only has access to reviews.)

© 2023. Published by The Company of Biologists under the terms of the Creative Commons Attribution License (https://creativecommons.org/licenses/by/4.0/).

As you will see, the reviewers gave favourable reports but reviewer 2 has made some suggestions regarding the text that you might like to consider. It is easier for you to make these changes now rather than at the proof stage hence sending the MS back to you before I formally accept your paper.

Please ensure that you clearly highlight all changes made in the revised manuscript. Please avoid using 'Tracked changes' in Word files as these are lost in PDF conversion.

I should be grateful if you would also provide a point-by-point response detailing how you have dealt with the points raised by the reviewers in the 'Response to Reviewers' box. Please attend to all of the reviewers' comments. If you do not agree with any of their criticisms or suggestions please explain clearly why this is so.

Reviewer 1

Advance summary and potential significance to field

The authors present a revised version of their manuscript describing Fast4DReg, a tool for the popular FIJI platform for the correction of axial and lateral drift in time-lapse imaging datasets.

Comments for the author

I thank the authors for their detailed responses to the points I raised previously and especially for their efforts in producing additional figures illustrating the use of Fast4DReg on other datasets - great work.

I am satisfied that all the points I raised previously have been addressed.
I'm sure Fast4DReg will prove to be a very popular tool among microscopists - I will undoubtedly be making use of it myself and recommending it to colleagues.

Best,

Dave Barry
Francis Crick Institute.

Reviewer 2

Advance summary and potential significance to field

I would like to thank the authors for the updated version of the draft. My earlier recommendations and question were answered nicely. I in particular value the explanation of the multi-channel image alignment (lines 170 and following). It is now very clear, well explained and the limitations listed. Well done!

Comments for the author

I have only minor suggestions. E.g. I propose to change a word here and there. As I'm not a native English speaker, feel free to ignore those suggestions in case they appear not reasonable.

114: "very noisy": please specify an approximate SNR 115: Consider using "maximum intensity projections" instead of "maximal intensity projections"

157 and following: Consider using "multichannel" instead of "multicolor"
165 and 167: Consider using "successfully" instead of "efficiently"
220 and 225: CCM is introduced twice. Once as "cross-correlation matrix" and once as "cross-correlation map". I recommend sychronizing these two.
235: Consider "memory-saving" instead of "RAM-saving" as "RAM" wasn't introduced before.
370: "mean" instead of "means"

© 2023. Published by The Company of Biologists under the terms of the Creative Commons Attribution License (https://creativecommons.org/licenses/by/4.0/).

373: consider "pixel intensity" instead of "pixels"
532 and 547: consider "intensity projection along Y" instead of "y-projection" and "Y-projection"
Fig 2D and 3F: Consider using "PCC" instead of "Pearson's correlation coefficient"
Fig 3E: The fourth panel (SNR 1.2) looks like a misaligned slice (which happens in low-SNR image registration). The figure caption could mention/explain why this is orange/red and not mostly white like all other panels.

Best regards,
Robert Haase
robert.haase@tu-dresden.de

Second revision

<u>Author response to reviewers' comments</u>

We thank both reviewers for taking the time to assess our revised manuscript and for recommending publication.

Reviewer 1 Advance Summary and Potential Significance to Field:
The authors present a revised version of their manuscript describing Fast4DReg, a tool for the popular FIJI platform for the correction of axial and lateral drift in time-lapse imaging datasets.

Reviewer 1 Comments for the Author:
I thank the authors for their detailed responses to the points I raised previously and especially for their efforts in producing additional figures illustrating the use of Fast4DReg on other datasets - great work.

I am satisfied that all the points I raised previously have been addressed.

I'm sure Fast4DReg will prove to be a very popular tool among microscopists - I will undoubtedly be making use of it myself and recommending it to colleagues.

Reviewer 2 Advance Summary and Potential Significance to Field:
I would like to thank the authors for the updated version of the draft. My earlier recommendations and question were answered nicely. I in particular value the explanation of the multi-channel image alignment (lines 170 and following). It is now very clear, well explained and the limitations listed. Well done!

Reviewer 2 Comments for the Author:
I have only minor suggestions. E.g. I propose to change a word here and there.
As I'm not a native English speaker, feel free to ignore those suggestions in case they appear not reasonable.

114: "very noisy": please specify an approximate SNR
Done

115: Consider using "maximum intensity projections" instead of "maximal intensity projections"
Done

157 and following: Consider using "multichannel" instead of "multicolor"
Done

165 and 167: Consider using "successfully" instead of "efficiently"
Done

220 and 225: CCM is introduced twice. Once as "cross-correlation matrix" and once as "cross-

© 2023. Published by The Company of Biologists under the terms of the Creative Commons Attribution License (https://creativecommons.org/licenses/by/4.0/).

correlation map". I recommend sychronizing these two.
Done

235: Consider "memory-saving" instead of "RAM-saving" as "RAM" wasn't introduced before.
Done

370: "mean" instead of "means"
Done

373: consider "pixel intensity" instead of "pixels"
Done

532 and 547: consider "intensity projection along Y" instead of "y-projection" and "Y-projection"
We kept y-projection so that our nomenclature remains consistent.

Fig 2D and 3F: Consider using "PCC" instead of "Pearson's correlation coefficient"
Done

Fig 3E: The fourth panel (SNR 1.2) looks like a misaligned slice (which happens in low-SNR image registration). The figure caption could mention/explain why this is orange/red and not mostly white like all other panels.
Done

Third decision letter

MS ID#: JOCES/2022/260728

MS TITLE: Fast4DReg: Fast registration of 4D microscopy datasets

AUTHORS: Joanna Pylvänäinen, Romain F Laine, Bruno MS Saraiva, Sujan Ghimire, Gautier Follain, Ricardo Henriques, and Guillaume Jacquemet
ARTICLE TYPE: Tools and Resources

I am happy to tell you that your manuscript has been accepted for publication in Journal of Cell Science, pending standard ethics checks.

© 2023. Published by The Company of Biologists under the terms of the Creative Commons Attribution License (https://creativecommons.org/licenses/by/4.0/).
